# Cyclin‐dependent kinase 13 is indispensable for normal mouse heart development

**DOI:** 10.1111/joa.14175

**Published:** 2024-11-18

**Authors:** Qazi Waheed‐Ullah, Anna Wilsdon, Aseel Abbad, Sophie Rochette, Frances Bu'Lock, Asma Ali Saed, Marc‐Phillip Hitz, J. David Brook, Siobhan Loughna

**Affiliations:** ^1^ School of Life Sciences, Faculty of Medicine and Health Sciences University of Nottingham Nottingham UK; ^2^ East Midlands Congenital Heart Centre University Hospitals of Leicester NHS Trust Leicester UK; ^3^ Institute of Medical Genetics Carl von Ossietzky University Oldenburg Oldenburg Germany; ^4^ Department of Congenital Heart Disease and Paediatric Cardiology University Hospital of Schleswig‐Holstein Kiel Germany; ^5^ German Centre for Cardiovascular Research (DZHK) Kiel Germany

**Keywords:** *Cdk13*, CHD, congenital heart disease, congenital heart disorders, cyclin‐dependant kinase 13, high‐resolution episcopic microscopy

## Abstract

Congenital heart disease (CHD) has an incidence of approximately 1%. Over the last decade, sequencing studies including large cohorts of individuals with CHD have begun to unravel the genetic mechanisms underpinning CHD. This includes the identification of variants in *cyclin‐dependent kinase 13* (*CDK13*), in individuals with syndromic CHD. CDK13 encodes a serine/threonine protein kinase. The cyclin partner of CDK13 is cyclin K; this complex is thought to be important in transcription and RNA processing. Pathogenic variants in *CDK13* cause CDK13‐related disorder in humans, characterised by intellectual disability and developmental delay, recognisable facial features, feeding difficulties and structural brain defects, with 35% of individuals having CHD. To obtain a greater understanding for the role that this essential protein kinase plays in embryonic heart development, we have analysed a presumed loss of function *Cdk13* transgenic mouse model (*Cdk13* ^*tm1b*^). The homozygous mutants were embryonically lethal in most cases by E15.5. X‐gal staining showed *Cdk13* expression localised to developing facial regions, heart and surrounding areas at E10.5, whereas at E12.5, it was more widely present. In the E15.5 heart, staining was seen throughout. RT‐qPCR showed significant reduction in *Cdk13* transcript expression in homozygous compared with WT and heterozygous hearts at E10.5 and E12.5. Detailed morphological 3D analysis of embryonic and postnatal hearts was performed using high‐resolution episcopic microscopy, which affords a more detailed analysis of structures such as cardiac valve leaflets and endocardial cushions, compared with more traditional histological techniques. We show that both the homozygous and heterozygous *Cdk13* ^*tm1b*^ mutants exhibit a range of CHD, including ventricular septal defects, bicuspid aortic valve, double outlet right ventricle and atrioventricular septal defects. 100% (*n* = 4) of homozygous hearts displayed CHD. Differential expression was seen in *Cdk13* ^*tm1b*^ homozygous mutants for two genes known to be necessary for normal heart development. The types of defects, and the presence of CHD in heterozygous mice (17.02%, *n* = 8/47), are consistent with the CDK13‐related disorder phenotype in humans. This study provides important insights into the effects of reduced function of *CDK13* in the mouse heart and contributes to our understanding of the mechanism behind this disorder as a cause of CHD.

## Graphical abstract

Congenital heart disease (CHD) is the most common defect in live births. The role of cyclin‐dependent kinase (CDK13) in cardiogenesis and CHD was studied using a transgenic mouse model (*Cdk13* ^*tm1b*^) carrying deletion of exons 3 and 4, causing loss of function. High‐resolution episcopic microscopy (HREM) allows detailed morphological analysis to be performed. A range of CHDs were found in the embryonic day E15.5 mouse heart, in both *Cdk13* ^*tm1b*^ heterozygotes and homozygotes. Ao, aorta; IVS; interventricular septum; LA, left atrium; LV, left ventricle; PT, pulmonary trunk; RA, right atrium; RV, right ventricle.

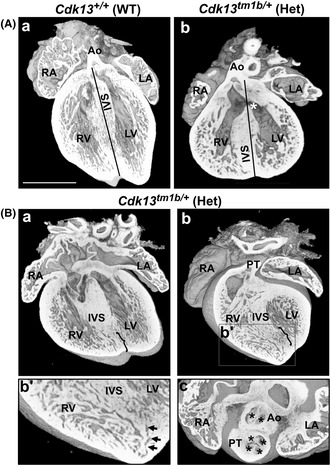

## 1 INTRODUCTION

Congenital heart disease (CHD) is a structural defect that occurs due to a disruption to the normal morphogenesis of the heart. Collectively, it is the most common congenital anomaly and has a higher associated mortality than many other congenital defects, representing a major health issue (Ossa Galvis et al., 2023). The majority of CHD cases are associated with a genetic cause. This can include chromosomal or structural variants, as well as sequence variants. Exome sequencing in large CHD cohorts has been used to understand better the genetic contributions (Homsy et al., 2015; Jin et al., 2017; Richter et al., 2020; Sifrim et al., 2016; Zaidi et al., 2013), with variants in some genes significantly over‐represented in syndromic CHD (having a distinct facial gestalt or at least one extra‐cardiac malformation) (Sifrim et al., 2016). In non‐syndromic (isolated) CHD, there was evidence of reduced penetrance of seemingly deleterious variants in known CHD genes. One novel gene associated with CHD, recently identified by our group, was *CDK13* (Sifrim et al., 2016).

Cyclin‐dependent kinase 13 (*CDK13*; also known as *CDC2L5*, *CHED*, *CDC2L* and *KIAA1791*) encodes a serine–threonine protein kinase. It is formed of 1512 amino acids, with a centrally located kinase domain. The cyclin partner for CDK13 is cyclin K. The active CDK13‐cyclin K complex is thought to play a role in the regulation of transcription and RNA processing (Chepelev, 2012; Even et al., 2016; Liang et al., 2015). To date, research on CDK13 function has focussed on its role in cancer; amplification of *CDK13* has been seen in tumour cells (Kim et al., 2012) and *CDK13* RNA over‐editing is a marker of poor prognosis in certain cancers (Dong et al., 2018).

Pathogenic variants in *CDK13* cause CDK13‐related disorder. This is characterised by intellectual disability, developmental delay, dysmorphic facial features, feeding difficulties and structural brain and heart defects (Hamilton & Suri, 2019). Pathogenic variants are mainly clustered within the kinase domain, emphasising the critical role of this domain in CDK13 function (Hamilton & Suri, 2019). A total of 91 different pathogenic and likely pathogenic variants in 172 affected individuals have been reported in the literature (Sifrim et al., 2016, Hamilton et al., 2018, Trinh et al., 2019, Bostwick et al., 2017, Uehara et al., 2018, van den Akker et al., 2018, Yakubov et al., 2019, Morison et al., 2023, Rouxel et al., 2022, Carneiro et al., 2018, Cui et al., 2022, Acharya et al., 2021, Gibbs et al., 2023, Cui et al., 2022, (Kacpura & Rodriguez‐Buritica, 2021, Wang et al., 2020), in the public repositories ClinVar (Landrum et al., 2018) and Decipher (Firth et al., 2009). The majority of these variants were heterozygous, and only a single consanguineous family with homozygous missense variants has been described (Acharya et al., 2021). Reported variants included missense, frameshift, truncating and splice‐site variants. Fifty‐three of these 91 variants (58.2%) were located in the kinase domain. In patients with a reported phenotype (138/172), CHD was present in 42% (58/138). A single common missense variant was seen (p.N842S), occurring in 48 patients, with CHD affecting 52.1% (*n* = 25) of this group.

In this study, we demonstrate that *CDK13* is essential for cardiogenesis. This had been indicated by a previous analysis of a hypomorphic *CDK13* mouse model, where homozygous embryos died mid‐gestation, possibly due to cardiac insufficiency (Nováková et al., 2019). To obtain a greater understanding for the role that this essential protein kinase plays in normal embryonic heart development and in the formation of structural heart defects, we analysed a *Cdk13* transgenic mouse model (*Cdk13* ^*tm1b*^). This mouse model carries a deletion of exons 3 and 4, which results in a premature stop codon prior to the critical functional domains, causing loss of function. We characterised this *Cdk13* transgenic mouse model by high‐resolution episcopic microscopy (HREM) to determine the types of CHD at E15.5 and in the postnatal day 6 heart. In addition, we analysed *Cdk13* expression and potential downstream targets of *Cdk13* and performed an assessment of lethality.

## 2 MATERIALS AND METHODS

### 2.1 Animal maintenance and genotyping

Studies were conducted in accordance with UK Home Office Animals (Scientific Procedures) Act (1986), under project license PPL PP5876461. *Cdk13* ^*tm1b(EUCOMM)Hmgu*^ mice were obtained from Wellcome Trust Sanger Institute which carried a Cre‐mediated excision of the parental *Cdk13* ^*tm1a(EUCOMM)Hmgu*^ allele resulting in the removal of the promoter‐driven neomycin selection cassette, and critical exons 3 and 4, leaving behind the inserted *lacZ* reporter sequence (see Figure S1). Wild‐type mice (C57BL/6N) were obtained from Charles River. Heterozygous mice for *Cdk13* ^*tm1b*^ (*Cdk13* ^*tm1b/+*^) were backcrossed with wild‐type (WT; *Cdk13* ^*+/+*^) mice for colony maintenance, and with heterozygous mice for obtaining homozygous (*Cdk13* ^*tm1b/tm1b*^) embryos. The day of visualising a vaginal plug was considered as E0.5. The day of birth was considered as P0.

Genotyping was done by end point PCR. The thermal cycler conditions comprised of 5‐min polymerase activation at 94°C and 30 cycles of 94°C, 30 s; 58°C, 30 s; and 72°C, 1 min. Primer sequences are provided in Table 1 of the supplementary information—Data S1.

**TABLE 1:** Genotype distribution of *Cdk13* ^*tm1b*^ embryos from heterozygous × heterozygous crosses at E12.5 and E15.5.

	WT	Het	Hom	Chi‐square test
E12.5				
Expected	66.75 (25%)	133.5 (50%)	66.75 (25%)	*p* = 0.0000055
Observed	70 (26.2%)	164 (61.4%)	33 (12.4%)	
E15.5				
Expected	35.25 (25%)	70.5 (50%)	35.25 (25%)	*p* = 0.0000002
Observed	52 (36.9%)	81 (57.4%)	8 (5.7%)	
Pearson's chi‐square test		*p* = 0.0196		

Abbreviations: Het, heterozygous; Hom, homozygous; WT, wild type.

### 2.2 Measurement of CRL and tissue collection

All animals were humanely culled. Embryos were collected at E10.5, E12.5 and E15.5. Crown‐rump length (CRL) of the collected embryos was measured as the maximum distance between their cephalic and their caudal poles (Mu et al., 2008). Embryos and postnatal Day 6 (P6) pups were dissected using a stereo microscope (Zeiss Discovery V8) and staged (Kaufman, 1995). For HREM, E15.5 and P6 hearts were isolated and washed in PBS (37°C), fixed in 4% PFA (37°C) for 20 min, washed in distilled water (37°C) for less than an hour until clear of blood, then fixed in 4% PFA (4°C) overnight. For qPCR and X‐gal staining experiments, embryos were dissected in 1X DEPC‐PBS (4°C). For qPCR, hearts were snap frozen in liquid nitrogen and stored at −80°C. The embryos for X‐gal staining were fixed in 3.7% PFA (in 1X DEPC‐PBS, 4°C) for 20–50 min before further processing.

### 2.3 RNA extraction and RT‐qPCR

Total RNA was extracted from E10.5 and E12.5 hearts using RNeasy Micro kit (Qiagen Cat. # 74004) and treated with RNase‐free DNase I provided in the kit following the manufacturer's protocol. The quality and quantity of extracted RNA was assessed using NanoDrop™ spectrophotometer (ND1000, Thermo Fisher Scientific®). A 260/280 of ~2.0 was considered pure. 500 ng of RNA was reverse transcribed using SuperScript™ II Reverse Transcriptase kit with random hexamers (Invitrogen Cat. # 18064014) following the manufacturer's protocol, then diluted five times with nuclease‐free water. A no‐reverse transcriptase reaction (RT‐) was included for each sample to assess for gDNA contamination. SYBR green dye (iTaq Universal SYBR Green Supermix BIO‐RAD Cat. # 1725121) was used for the qPCR reactions. To determine primer efficiencies, generate melt curves and determine the best dilution to use for qPCR, a standard curve was generated with six dilution points of 1:3 from stock cDNA. Efficiencies between 85% and 110% were accepted, with *R* ^2^ values ≥0.98. For relative quantification, three biological and three technical replicates, a no‐template control (NTC) and RT‐ controls were included in each plate. The reaction mix (10 μL) contained 1 μL of stock cDNA, 250 nM of reverse and forward primers and 5 μL of SYBR green. *Pgk1* and *Rpl4* were used as reference genes. Reactions were run on Applied Biosystems® 7500 fast real‐time PCR system. The thermocycler conditions were similar for all reactions and comprised of 50°C, 2 min; 95°C, 1 min; 40 cycles of 95°C, 15 s; and 60°C, 15 s; followed by melt curve stage that comprised of 94°C, 15 s; 60°C, 1 min; 95°C, 30 s; and 60°C, 15 s. Primers for qPCR were designed and checked for specificity using primer3 and BLAST (Koressaar & Remm, 2007; Untergasser et al., 2012; Ye et al., 2012). The primer sequences for *Cdk13* and the cardiac‐specific genes *Sall4*, *Vegfa*, *Edn1*, *Ednra* and *Eln* are in Table S1. Primers for reference genes (Ruiz‐Villalba et al., 2017) were as previously described (Waheed‐Ullah et al., 2024) (Table S1). PCR products were run on 2% agarose gel to check for the correct amplicon length.

### 2.4 Hearts processing for HREM

A detailed protocol has been provided for HREM previously (Waheed‐Ullah et al., 2024). In summary, hearts previously fixed in 4% PFA were washed in PBS, then dehydrated with increasing concentrations of methanol (from 10% to 100%) and embedded in JB4 methacrylate resin containing eosin and acridine orange (Sigma Aldrich EM0100‐1KT) as described previously (Weninger et al., 2018). Briefly, the samples were immersed in a 50:50 mix of 100% methanol:JB4 dye mix overnight, and then in pure JB4 dye mix overnight. The hearts were then placed in embedding blocks containing polymerising JB4 dye mix, and left overnight to solidify at room temperature, and stored at 4°C. Before sectioning, hearts were baked at 95°C for 24 h and then kept at 4°C for 24 h. E15.5 and P6 hearts were sectioned at 2 and 3 μm thickness, respectively, using high‐resolution episcopic microscopy (HREM) (Indigo Scientific®). Images of block surface were captured by JENOPTIK GRYPHAX® ProgRes® microscope camera. The z stack of images obtained after sectioning was downsized, cropped and inverted using GraphicConverter® v9 (Lemkesoft, Germany), ImageJ® v1.52 and Adobe Photoshop® CC 2019 (version 20.0), and were then visualised in three dimensions using OsiriXä 11.0 (Pixmeo SARL, Switzerland).

### 2.5 X‐gal staining and sectioning

The protocol used was adapted from those previously reported (Gierut et al., 2014; Loughna & Henderson, 2007). The previously fixed embryos were permeabilised using rinse buffer. The rinse buffer was prepared in DEPC‐PBS using sodium phosphate dibasic (0.5 M; pH 7.0–7.5), sodium phosphate monobasic (0.5 M; pH 7.0–7.5), magnesium chloride (1 M), sodium deoxycholate (1.5 mM) and octylphenoxypolyethoxyethanol (IGEPAL CA‐630, 3%; Cat no. I8896, Sigma‐Aldrich®). Rinse buffer was added to the embryos and left overnight at 4°C on rocker. X‐gal staining was carried out the next day at 37°C in incubator overnight using freshly prepared X‐gal staining solution (1 mg/mL), prepared from 20 mg/mL X‐gal stock (Thermo Scientific™ Cat. # R0941) in 3 mM rinse buffer. The next day, samples were rinsed with rinse buffer, and then fixed in 3.7% PFA (in 1X DEPC‐PBS) at 4°C overnight. Thereafter, the samples were placed in 50% glycerol (prepared in 1X PBS) at 4°C overnight for imaging next day. Imaging was performed by using microscopes (ZEISS Stemi SV11 and ZEISS SteREO Discovery.V8) with attached camera.

The E15.5 stained heart was embedded using an adapted version of a previously described protocol (Blanco et al., 2018). The heart was washed in PBS and then dH_2_O before dehydration in a graded ethanol series to 100% ethanol. Ethanol was then replaced with 100% isopropanol for 30 min at room temperature. The heart was then incubated in pre‐warmed isopropanol and incubated at 60°C for 30 min, and then transferred to a pre‐warmed 50% isopropanol/50% paraffin wax solution and incubated for 4 h at 60°C. It was immersed in 60°C paraffin with two wax changes of 30 min each prior to embedding. Paraffin blocks were sectioned at 10 μm. For deparaffinisation, slides were incubated at 60°C for 45 min and then immersed in isopropanol at 60°C for 5 min, followed by 3 min at room temperature. Rehydration was carried out by incubating in an ethanol series at room temperature, and then rinsed twice with dH_2_O. Counterstaining was performed using 1% eosin for 5 min at room temperature, followed by washing in dH_2_O for 1 min. After staining, sections were dehydrated in 95% ethanol twice for 2 min each, 100% ethanol twice for 2 min each and briefly washed in xylene. Slides were mounted with DPX mounting medium and cover slipped for microscopy. Imaging was performed using a ZEISS Axioscan 7 slide scanner.

### 2.6 Data analysis

HREM‐sectioned hearts were initially evaluated being blind to the genotype, independently by two researchers, followed by group analysis with four researchers for detailed morphological assessment. Reference genes for qPCR were validated using Refinder (http://blooge.cn/RefFinder/). qPCR standard curves and melt curves were examined using software for7500 fast real‐time PCR system (Applied Biosystems™) v2.0.6.

Comparative qPCR data were analysed using a modified Pfaffl method to normalise to two reference genes as described previously (Hellemans et al., 2007; Vandesompele et al., 2002). In case of negative control amplification, a difference of 10 Cq values or more between the RT‐ or NTC and the corresponding RT+ sample was accepted. For quantitative data, one‐way ANOVA (*p* ≤ 0.05) was applied using GraphPad Prism 9.2.0 to compare the three groups followed by post hoc Tukey (multiple comparisons) test. For comparing two groups, unpaired *t*‐test (*p* ≤ 0.05) was applied. To carry out lethality assessment, chi‐squared test and Pearson chi‐squared test (*p* ≤ 0.05) were applied using MS‐Excel.

## 3 RESULTS

### 3.1 Increased embryonic lethality and reduced *Cdk13* expression in the *Cdk13* ^*tm1b*^ homozygous heart

**FIGURE 1:**
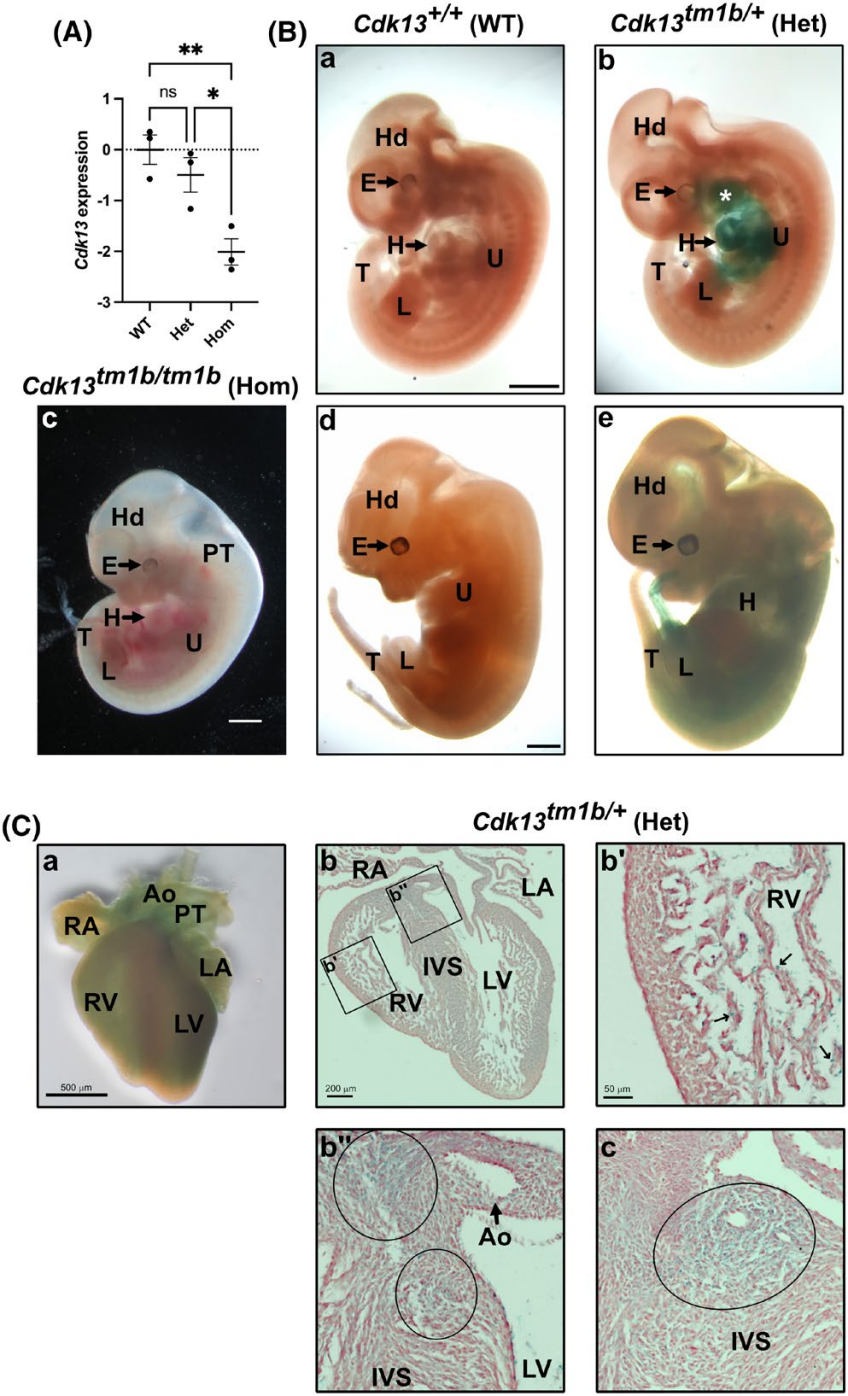
Expression of *Cdk13* in *Cdk13* ^*tm1b*^ mouse line. (A) Expression in E12.5 mouse hearts by RT‐qPCR. The log_2_FC of *Cdk13* (mean with SEM) in heterozygous (Het; *Cdk13* ^*tm1b/+*^) and homozygous (Hom; *Cdk13* ^*tm1b/tm1b*^) hearts collected at E12.5 compared to WT (*Cdk13* ^*+/+*^). Using one way‐ANOVA and post hoc Tukey test, no statistically significant difference is present in the level of *Cdk13* transcript in heterozygous compared to WT (*p* = 0.5049) hearts. In contrast, the difference is significant in homozygous compared to WT (*p* = 0.0073) and heterozygous (*p* = 0.0263) hearts. Het, heterozygous; Hom, homozygous; ns, not significant; WT, wild type. (B) X‐gal staining to show *Cdk13* expression in *Cdk13* ^*tm1b/+*^ (Het) embryos. At E10.5, in comparison to WT control (a), X‐gal staining is localised to the developing facial regions (asterisk denotes maxillary and mandibular processes of 1st pharyngeal arch), and the heart (H) and surrounding area (b). A whole E12.5 embryo is shown (c) to allow comparison to WT (d) and heterozygous (e); note that the WT and heterozygous are littermates, but the homozygous is not. At E12.5, X‐gal staining is more widely present with staining seen to the heart (H) and abdominal regions, and faint staining to the head (d). X‐gal staining was not seen in WT control (c). To visualise the heart, the upper limb in (d) has been removed. E, eye; H, heart; Hd, head; Het, heterozygous; U, upper limb bud; L, lower limb bud; T, tail; WT, wild type. Scale in a, c and d 1 mm, same magnifications in b and d respectively. (C) Anterior view of a E15.5 X‐gal stained *Cdk13* ^*tm1b/+*^ (Het) heart shows widespread staining (a). Upon sectioning, expression could be seen to all regions (b), with noticeably punctate staining in the trabeculae of the ventricular chambers (b'). Slightly more intense areas of staining were seen at the superior aspect of the interventricular septum (within small and large circles in b″). Another section also shows this more intense staining at the superior aspect of the interventricular septum (denoted in oval in c); two lumens of the left coronary artery branches can be seen within. Ao, aorta; IVS, interventricular septum; LA, left atrium; LV, left ventricle; PT, pulmonary trunk; RA, right atrium; RV, right ventricle. Scale bar: A, 500 μm; b, 200 μm; b', 50 μm (same magnification for b″ and c).

Genotype distribution of E12.5 and E15.5 embryos from *CDK13* ^*TM1B*^ heterozygous crosses was determined (Table 1). At E12.5, of the total 267 embryos collected, 70 (26.2%) were WT, 164 (61.4%) heterozygous and 33 (12.4%) homozygous. At E15.5, of the total 141 embryos collected, 52 (36.9%) were WT, 81 (57.4%) were heterozygous and 8 (5.7%) were homozygous. on lethality assessment, based on genotype of embryos collected, we found that the observed ratios of homozygotes were less than the expected mendelian ratios and that this difference was statistically significant at both stages (chi‐squared test, *P* = 0.0000055 and *P* = 0.0000002, respectively). comparing E12.5 with E15.5 in terms of expected mendelian ratios, significantly less homozygotes were obtained at E15.5 compared with E12.5 (Pearson's chi‐squared test, *p* = 0.0196). After breeding and genotyping 866 mice from this colony, live‐born homozygous *CDK13* ^*TM1B*^ pups were not obtained. This indicates that loss of both alleles of *CDK13* is mostly embryonically lethal by E15.5 and is not compatible with extrauterine life, although later than E15.5, embryonic stages were not analysed. External phenotypic analysis of the heterozygous embryos (*N* = 245) shows that pericardial effusion was present in 4 (1/164 at E12.5 and 3/81 at E15.5) and midfacial cleft (due to incomplete fusion of the medial nasal prominences) in one E12.5 embryo. In one E15.5 heterozygous embryo, right side anophthalmia and tongue protrusion, possibly due to mandibular hypoplasia, were noted. In homozygous embryos (*N* = 41), midfacial aplasia (a deficiency of midline facial structures) was seen in seven (5 at E12.5 and 2 at E15.5), pericardial effusion in four (3 at E12.5 and 1 at E15.5) and hydrocephalus in one E12.5 embryo. Growth retardation was present in eight heterozygous embryos (7/164 at E12.5 and 1/81 at E15.5) and 22 homozygous embryos (17/33 at E12.5 and 5/8 at E15.5). For comparison, a E12.5 homozygous embryo is shown in Figure 1Bc, compared to heterozygous and wild type (Figure 1Be,d, respectively) E12.5 embryos. A heartbeat was not seen in 4/164 E12.5 heterozygous embryos and 7/41 homozygous embryos (2/33 at E12.5 and 5/8 at E15.5). CRL measurements show that there was statistically significant reduction in CRL in homozygous compared to WT and heterozygous embryos at both E12.5 (*N* = 27, *P* = 0.0002, ANOVA) and E15.5 (*N* = 15, *p <* 0.0001, ANOVA) (Figure S2). No other defects were observed.

To determine if *CDK13* mRNA expression level was affected in the *CDK13* ^*TM1B*^ mouse embryonic heart, RT‐qPCR was performed on homozygous (*CDK13* ^*TM1B/TM1B*^), heterozygous (*CDK13* ^*TM1B /+*^) and WT (*CDK13* ^*+/+*^) hearts at E10.5 and E12.5. E10.5 and E12.5 were selected as the E10.5 heart is at an early stage of development (looping is in progress), and E12.5 when looping has completed but important processes, such as outflow tract and ventricular septation, are ongoing. Using one‐way ANOVA and post hoc Tukey test, there was a significant decrease in mRNA expression in the homozygous hearts, compared to both heterozygous and WT controls (*p* = 0.0263 and 0.0073, respectively, for E12.5, and *p* = 0.0330 and 0.0129, respectively, for E10.5) (Figure 1A and Figure S3). However, there was no significant difference in expression levels between heterozygotes and WT controls (*p* = 0.5049 at E12.5 and *p* = 0.7023 at E10.5).

### 3.2 X‐gal staining shows *Cdk13* expression is widely expressed in the mouse heart

To determine where in the embryo *Cdk13* is expressed, X‐gal staining was performed on mouse embryos. In *Cdk13* ^*tm1b/+*^ (heterozygous) E10.5 embryos, X‐gal staining was localised to the heart and the developing facial regions (Figure 1Bb). At E12.5, X‐gal staining was more widely present with intense staining seen to the thoracic and abdominal regions, and faint staining to the head (Figure 1Be). X‐gal staining was not seen in WT controls (Figure 1Ba,d). Staining was also seen throughout the *Cdk13* ^*tm1b/+*^ heart at E15.5 (Figure 1Ca). Sectioning of this heart showed low levels of staining to all regions (Figure 1Cb), with punctate expression seen to the trabeculae of the ventricular chambers (Figure 1Cb'). In addition, two slightly more intense areas of LacZ expression were to the superior aspect of the interventricular septum (denoted by a small and large circle in Figure 1Cb″); this is the region where fusion of the endocardial cushions with the components for ventricular septum has occurred. In a different more anterior section, expression could again be seen quite distinctly in this region (denoted by oval in Figure 1Cc).

### 3.3 Simple and complex congenital heart defects were seen in heterozygous mutant (*Cdk13* ^*tm1b/+*^) hearts

To determine if any structural defects were present in the *Cdk13* ^*tm1b*^ mutant heterozygous hearts, HREM was performed at E15.5. If cardiogenesis occurs normally, at this stage, a four‐chambered structure with correctly aligned outflow vessels can be seen, with the outflow tract and ventricular chambers having completed septation (Geyer et al., 2017). A total of 44 WT, 47 heterozygous and four homozygous embryos were analysed by HREM (Table 2).

**TABLE 2:** Congenital heart disease seen in E15.5 *Cdk13* ^tm1b^ mouse hearts.

Genotype	Total	*N* with CHD (%)	CHD phenotype
WT	44	0	‐
Het	47	8 (17%)	pMem VSD (outlet) pMem VSD (outlet) musc VSD BAV (two‐sinus type) DORV and *pMem VSD DORV and *pMem VSD pMem VSD (outlet), BAV (two‐sinus type) BAV (two‐sinus type), Quad PV, musc VSD
Hom	4	4 (100%)	AVSD, DORV, doubly committed subarterial inlet to outlet (confluent) single VSD AVSD, DORV, PS, inlet to outlet (confluent) single VSD, musc VSD AVSD, DORV, doubly committed subarterial inlet to outlet (confluent) single VSD, musc VSD AVSD, DORV, inlet to outlet (confluent) single VSD

*Note*: Each row indicates a separate embryo. *pMem VSD were single VSD with interconnected inlet and outlet components.

Abbreviations: Ao, aorta; AVSD, atrioventricular septal defect; BAV, bicuspid aortic valve; CHD, congenital heart disease; DORV, double outlet right ventricle; Het, heterozygous; Hom, homozygous; musc VSD, muscular ventricular septal defect; pMem VSD, perimembranous ventricular septal defect; PS, pulmonary stenosis; Quad PV, quadricuspid pulmonary valve; VSD, ventricular septal defect; WT, wild type.

**FIGURE 2:**
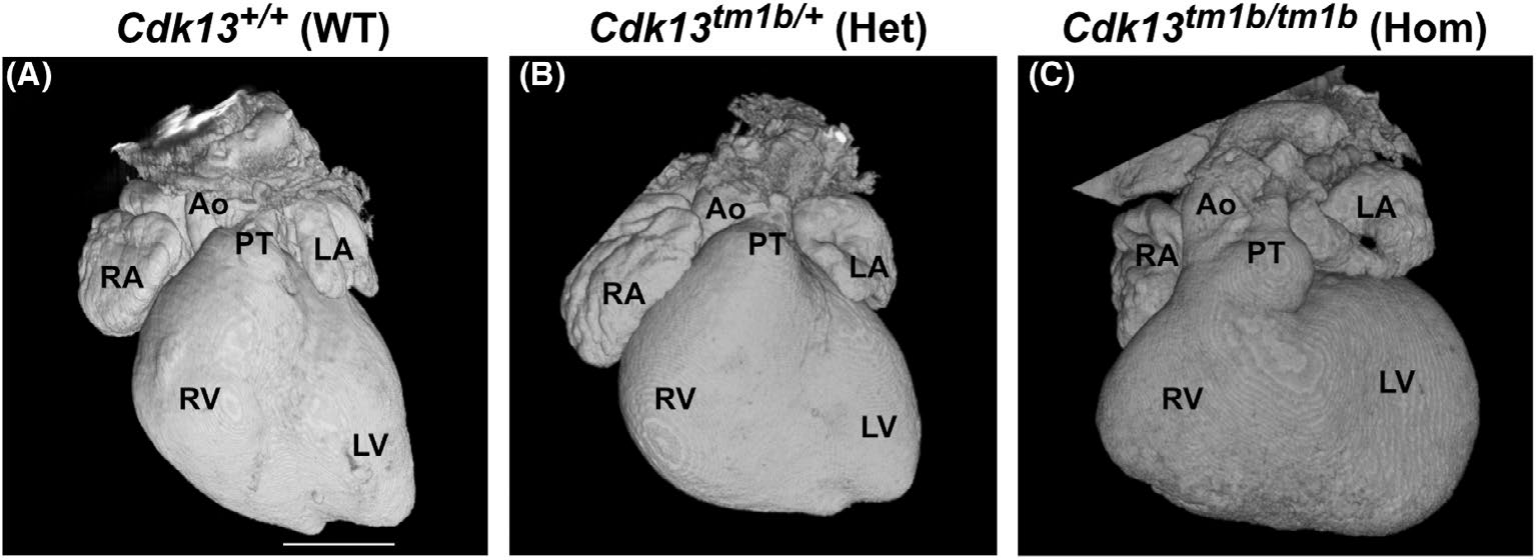
External analysis of E15.5 *Cdk13* ^*tm1b*^ homozygous, heterozygous and wild type hearts. (A–C): 3D reconstruction of wild type (a), heterozygous (b) and homozygous (c) whole hearts. Scale bar in (A) = 500 μm; same magnification for (B, C). Ao, aorta; Het; heterozygous; Hom, homozygous; LA, left atrium; LV, left ventricle; PT, pulmonary trunk; RA, right atrium; RV, right ventricle; WT, wild type.

**FIGURE 3:**
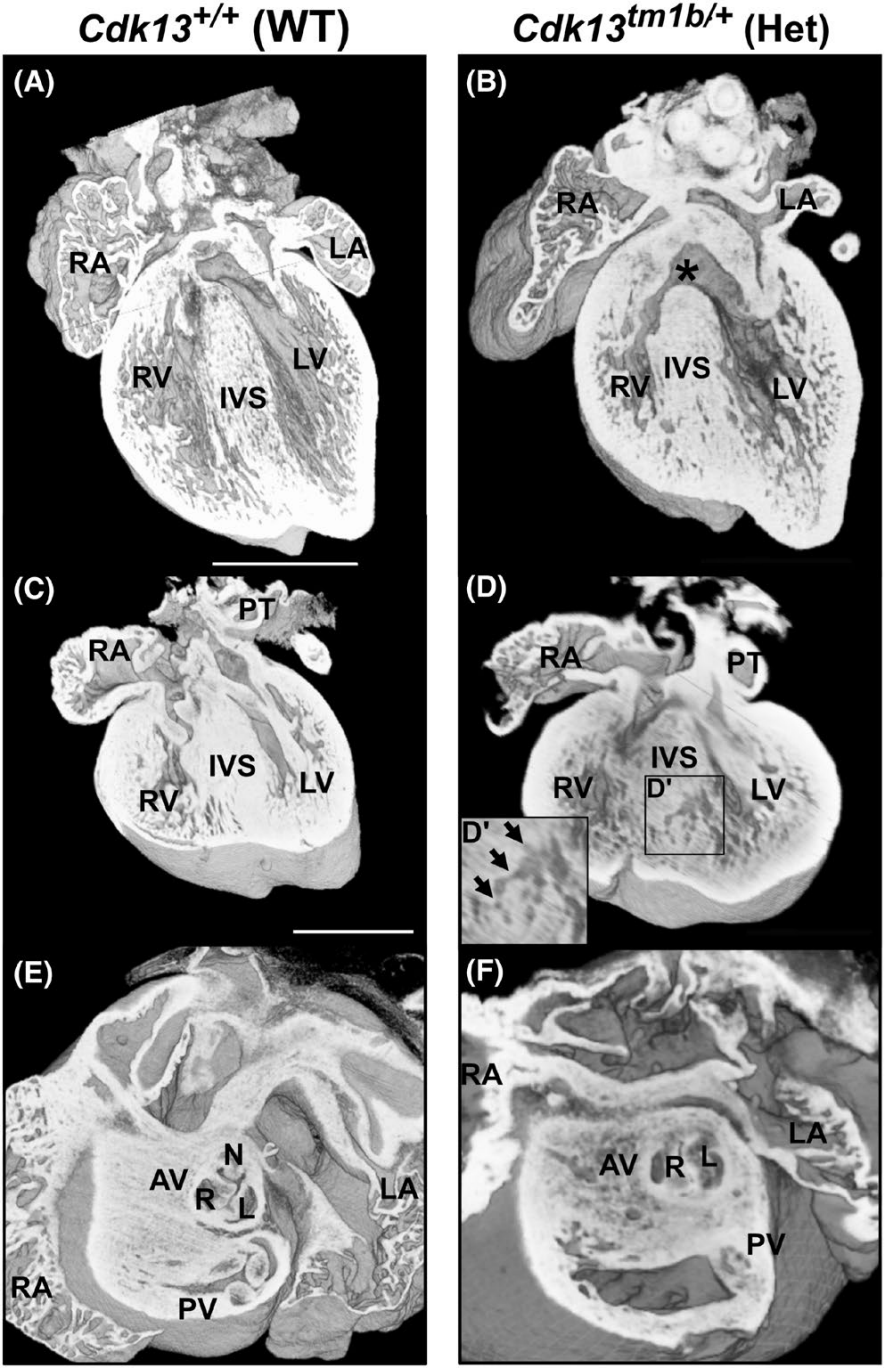
Isolated defects in heterozygous (*Cdk13* ^*tm1b/+*^) E15.5 mouse hearts. (A,B) Four chamber view of WT (*Cdk13* ^*+/+*^) heart (a) showing the left and right ventricular chambers separated by a complete interventricular septum (IVS). In comparison, a heterozygous (*Cdk13* ^***t**m1b/+*^) heart (b) has a perimembranous outlet ventricular septal defect (denoted by asterisk). The trabeculae also appear hypertrophic in comparison to the control heart in A. (C, D) Coronal section of WT (*Cdk13* ^*+/+*^) mouse heart (c) showing the left and right ventricular chambers separated from each other by an IVS in comparison to a heterozygous (*Cdk13* ^*tm1b /+*^) heart (D and inset D′) which had a muscular ventricular septal defect (denoted by black arrows). The myocardium also appears spongy in D compared to C. (E, F): Superior view of the WT heart (E) showing aortic valve having three leaflets and corresponding three sinuses—right (R), left (L) and non‐coronary (NC). In comparison, a heterozygous (*Cdk13* ^***t**m1b/+*^) heart (f) has bicuspid aortic valve, with just two leaflets and two sinuses. Scale bar in (A) = 500 μm; same magnification for (B). Scale bar in (C) = 500 μm; same magnification for (D). AV, aortic valve; Het; heterozygous; IVS, interventricular septum; L, left leaflet and sinus; LA, left atrium; LV, left ventricle; NC, non‐coronary leaflet and sinus; PT, pulmonary trunk; PV, pulmonary valve; R, right leaflet and sinus; RA, right atrium; RV, right ventricle; WT, wild type.

Of the 47 heterozygous hearts at E15.5, eight had CHD (17%). Externally, the hearts appeared similar to that of the WT embryo (Figure 2B compared to Figure 2A). None of the WT hearts had evidence of CHD (Table 2). Of the eight heterozygous hearts with structural heart abnormalities, four had simple defects (50%; Table 2; Figure 3). Three of these were ventricular septal defects (VSD; an opening between the right and left ventricular chambers). The VSDs were classified further. A perimembranous outlet VSD was present in two hearts (Figure 3B in comparison to WT shown in Figure 3A), and one heart displayed a muscular VSD (denoted by black arrows in Figure 3D,D'; compare to WT control in 3C) and spongy myocardium (Figure 3D). The trabeculae were also hypertrophic in two of these heterozygous hearts (Figure 3B and D)). In the fourth heterozygous heart with simple CHD, a bicuspid aortic valve (BAV) was identified (Figure 3F). In a normal heart, the three aortic valve leaflets are arranged as right, left and non‐coronary, as seen in control WT hearts (Figure 3E). However, this heterozygous heart with isolated BAV had two leaflets (the right and left leaflets) and two sinuses (Figure 3F).

**FIGURE 4:**
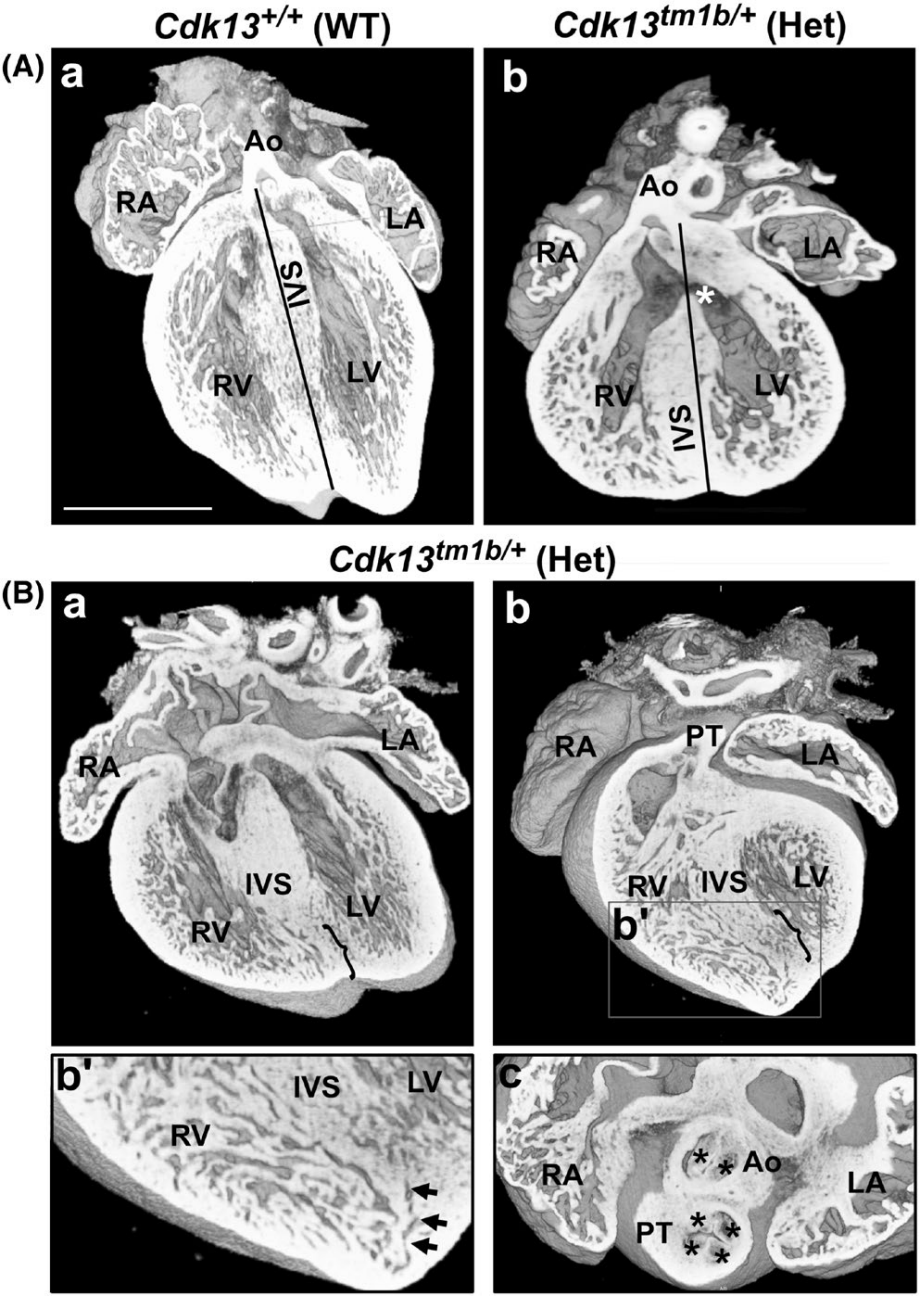
Complex defects are seen in heterozygous (*Cdk13* ^*tm1b /+*^) E15.5 mouse hearts. (A) A coronal section of E15.5 WT (*Cdk13* ^*+/+*^) mouse heart (a) showing the left and right ventricular chambers separated from each other by the interventricular septum (IVS), and the aorta (Ao) originating from left ventricle (LV), as denoted by the black line. In contrast, a coronal section of a heterozygous (*Cdk13* ^*tm1b/+*^) heart (b) shows double outlet right ventricle and a single interventricular communication (perimembranous ventricular septal defect, denoted by asterisk) with interconnected inlet and outlet components. The black line denotes that more than 50% of the diameter of the aorta connects to the RV instead of the LV. The trabeculae are also hypertrophic. Scale bar in a = 500 μm; same magnification for b. (B) A four‐chamber view of a heterozygous (*Cdk13* ^*tm1b/+*^) heart (a) appears to have recesses in the lower region of the ventricular septum (denoted by bracket). This can also be seen in a more ventral view of the same heart (b; see bracket). The trabeculae had a sponge‐like appearance. In addition, the boxed area (b') denotes a small muscular ventricular septal defect (shown by black arrows). This heart also had a two‐sinus bicuspid aortic valve (c; denoted by two black asterisks in aorta) and a quadricuspid pulmonary valve (c; denoted by 4 black asterisks in pulmonary trunk). Ao, aorta; LA, left atrium, LV, left ventricle; PT, pulmonary trunk; RA, right atrium; RV, right ventricle; IVS, interventricular septum. Scale bar in a = 500 μm; same magnification for b.

Of the four heterozygous hearts with more complex defects (Table 2), two had double outlet right ventricle (DORV) (Figure 4Ab, compared to control in Aa). In DORV, the aorta and the pulmonary trunk both arise completely or predominantly from the morphological right ventricle (Yim et al., 2018); in a normal heart, the aorta should arise from the left ventricle. There is usually an interventricular communication with DORV. In these two heterozygous hearts with DORV, both had a perimembranous VSD (denoted by asterisk in Figure 4Ab). The perimembranous VSDs in both hearts had interconnected inlet and outlet components (Table 2). In addition, the trabeculae were hypertrophic (Figure 4Ab).

A more complex set of defects was seen in the third of these four heterozygous hearts with complex CHD (Figure 4B). The ventricular septum appeared to have recesses in its inferior region and the trabeculae had a sponge‐like appearance (Figure 4Ba, Bb). In addition, at least one small muscular VSD could be seen within this region (Figure 4Bb', denoted by arrows). There were additional defects seen in the valves of the outflow vessels. The aortic valve was bicuspid with two sinuses, with the non‐coronary leaflet absent (Figure 4Bc). Furthermore, this same heart had four leaflets in the pulmonary valve, instead of the normal three (Figure 4Bc). The final heterozygous heart with complex CHD had a perimembranous outlet VSD and BAV, with two leaflets and two sinuses (Table 2).

### 3.4 Complex CHD was seen in all homozygous mutant (*Cdk13* ^*tm1b/tm1b*^) hearts

**FIGURE 5:**
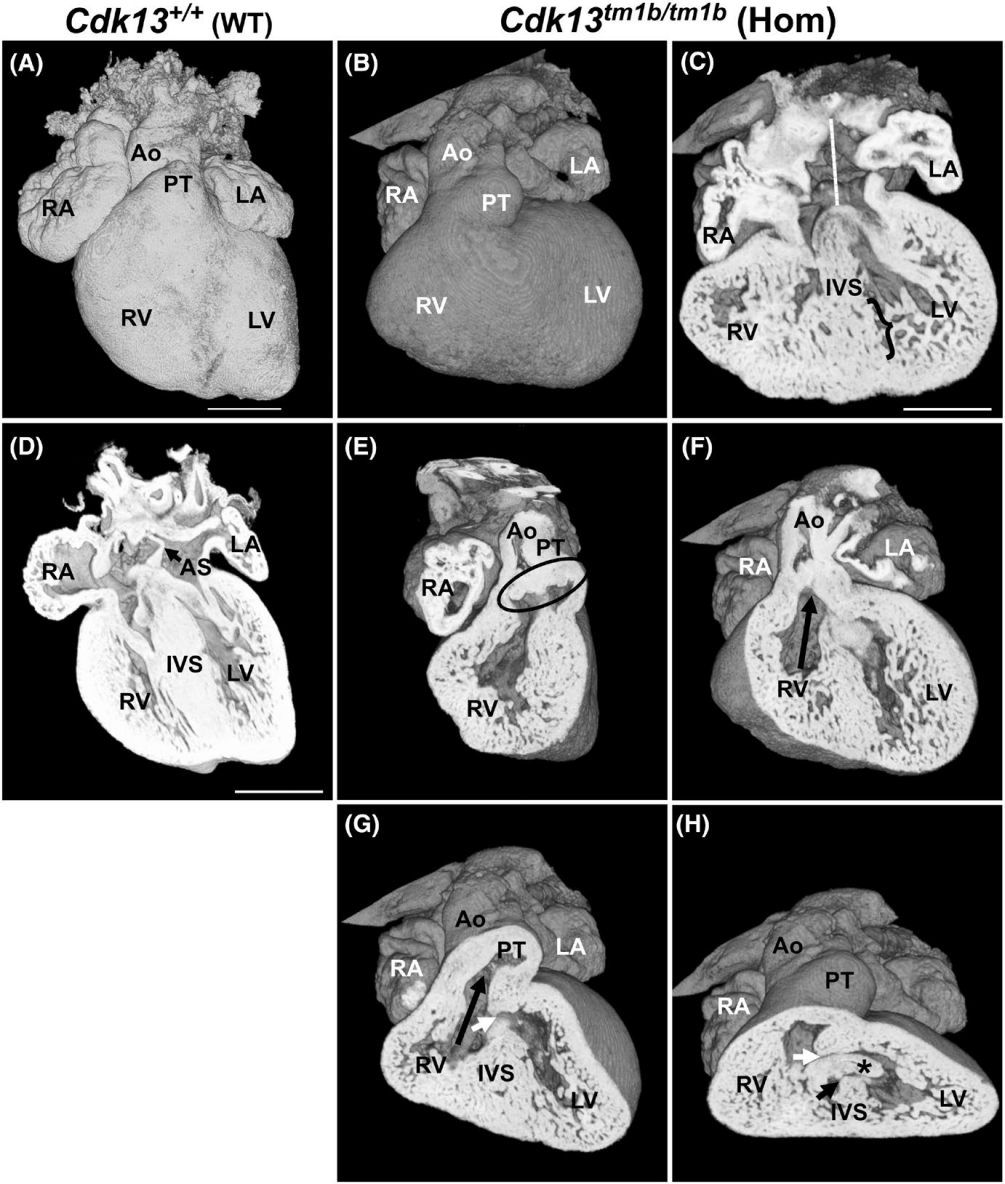
Homozygous (*Cdk13* ^*tm1b /tm1b*^) E15.5 heart with AVSD and DORV. Externally, this homozygous heart (B) has an abnormal shape with a rounded appearance compared with WT (A). Coronal view shows that the *Cdk13* ^*tm1b*^ homozygous heart (C) has AVSD with apparent absence of atrial septation (white line) in comparison to WT heart (D) where an atrial septum (AS) is present. The trabeculae are hypertrophic, and recesses are present in the interventricular septum (IVS) (C; denoted by bracket). A sagittal view from the right of the heart shows that there are aortic and pulmonary valve leaflets (encircled by black oval) present, but there is no underlying outlet septum (E); the expected number of leaflets were seen. In the homozygous heart, a ventral view shows both the aorta and pulmonary trunk arising from the right ventricle (F and G, respectively; denoted by black arrows). An outlet ventricular communication can be seen (G; denoted by small white arrow). Viewed in anteroinferior plane (H), the outlet (small white arrow) and inlet VSD (small black arrow) can both be seen and were found to be interconnected. The inlet ventricular communication (black arrow) is there as the ventral (*) endocardial cushion has failed to fuse with the dorsal endocardial cushion and muscular interventricular septum (IVS). Scale bar in (A) = 500 μm; same magnification for (B). Scale bar in (C, D) = 500 μm. Ao, aorta; AS, atrial septum; AVSD, atrioventricular septal defect; DORV, double outlet right ventricle; LA, left atrium; LV, left ventricle; PT, pulmonary trunk; RA, right atrium; RV, right ventricle; IVS, interventricular septum; VSD, ventricular septal defect.

The homozygous *Cdk13* ^*tm1b*^ mutant hearts were all abnormal externally, with a rounded (Figure 2C) or cobblestone appearance (Figure S4A) in comparison to both heterozygous and wild types (Figure 2A,B, respectively). The penetrance of defects in these four homozygous hearts analysed was 100%, with all having complex defects (Table 2; Figure 5 and Figure S4A). They all had AVSD (atrioventricular septal defect), DORV and a VSD (two had a single VSD with connected inlet and outlet components, and two had a doubly committed inlet to outlet VSD). One had pulmonary stenosis in addition (Figure S4A).

An AVSD forms when there is a failure of fusion between the primary atrial septum, dorsal mesenchymal protrusion (also known as the vestibular spine), mesenchymal cap or atrioventricular endocardial cushions (Burns et al., 2016; Taqatqa & Vettukattil, 2021; Webb et al., 1998). A common atrioventricular junction and valve are present (Anderson et al., 2010). At both the atrial and ventricular levels, the degree of septal deficiency and communication is highly variable (Franklin et al., 2017). One of these homozygous hearts with AVSD and DORV is shown in Figure 5. Externally, this homozygous heart was rounded (Figure 5B compared to WT in Figure 5A). Internal analysis shows that the atrial septal components were absent (denoted by white dotted line in Figure 5C) in comparison to controls, where the atrial septum can be seen (Figure 5D). Furthermore, there were deep recesses in the ventricular septum (denoted by bracketed region in Figure 5C; compared to the ventricular septum in a WT heart in Figure 5D) and the trabeculae were hypertrophic with a sponge‐like appearance. A more ventral view shows that both the aorta and pulmonary trunk arise from the right ventricle (Figure 5E,G). The aortic and pulmonary valve leaflets could be discerned in the outflow vessels and were otherwise normal (denoted by oval in Figure 5E). In addition, the interventricular communication was a doubly committed VSD with interconnected inlet and outlet components. The outlet VSD is shown in Figure 5G, and again from a more inferior view in Figure 5H (denoted by white arrows in both panels). The inlet VSD can be seen between the dorsal and ventral endocardial cushions, which have failed to fuse (Figure 5H, denoted by black arrow).

Further complex defects were seen in other homozygous hearts (Table 2). A representative heart is shown in Figure S4. These homozygous hearts had a cobblestone appearance on the external aspect (Figure S4A), possibly due to resorption as these embryos died before harvesting. Internal analysis shows that the atrial septal components were partly absent; the open arrow denotes some septal components in Figure S4B. The superior and inferior endocardial cushions had failed to fuse with the dorsal mesenchymal protrusion, resulting in an ostium primum defect (black arrow, Figure S4B) and hence an AVSD. In addition, the myocardial wall appeared deeply trabeculated and the ventricular septum appeared spongy with potentially several muscular VSDs (Figure S4B). Finally, this heart also had DORV with doubly committed subarterial single VSD with interconnected inlet and outlet components. Furthermore, the pulmonary trunk appears much smaller than the proximal aorta (Figure S3C,D), resulting in pulmonary arterial stenosis. However, both aortic and pulmonary valve leaflets appeared normal.

### 3.5 Abnormalities in heterozygous mutant (*Cdk13* ^*tm1b/+*^) hearts at neonatal stage P6

**FIGURE 6:**
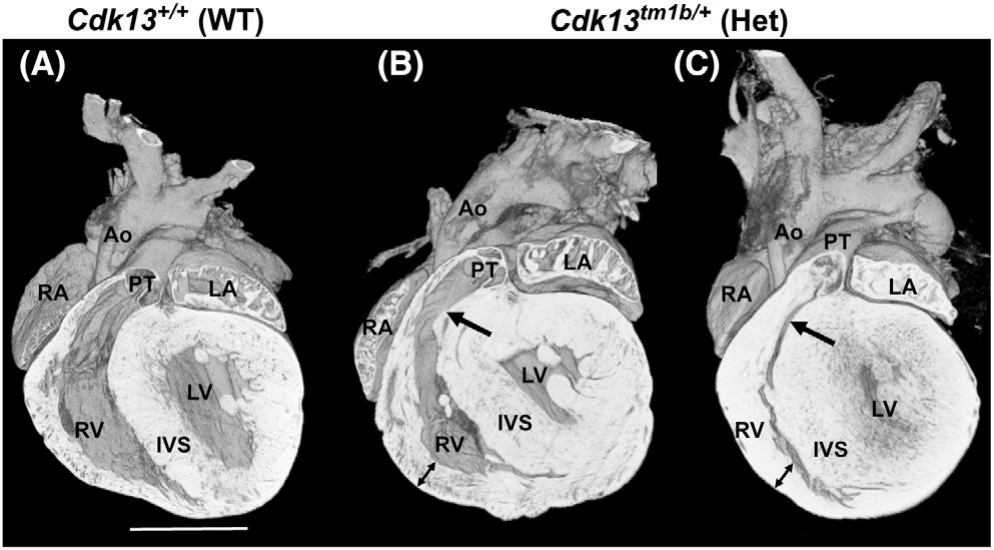
Increased ventricular wall thickness and narrowed right ventricle outflow region in P6 heterozygous (*Cdk13* ^*tm1b /+*^) hearts. Coronal sections of two P6 neonatal heterozygous (*Cdk13* ^*tm1b/+*^) hearts (B, C) with thickened ventricular walls (denoted by double headed arrows) compared to wild‐type control (A). Narrowing of the right ventricular outflow region (pulmonary trunk; PT) is denoted by a black arrow. Scale bar in (A) = 1 mm; same magnification for (B and C). Ao, aorta; LA, left atrium; LV, left ventricle; PT, pulmonary trunk; RA, right atrium; RV, right ventricle; IVS, interventricular ventricular septum; WT, wild type.

P6 hearts from *Cdk13* ^*tm1b*^ heterozygous (*n* = 9) mice were analysed by HREM and compared to WT (*n* = 6). In three of the nine heterozygous hearts analysed, there was right and left ventricular wall thickening, resulting in narrowing of the right ventricle outflow region into the pulmonary trunk (Figure 6B,C compared with wild‐type control A). In addition, in one P6 *Cdk13* ^*tm1b/+*^ heart, there were three coronary ostia in the aortic sinuses (Figure S5). There was one for the right coronary artery (RCA) in the right aortic sinus (black arrow), and two in the left coronary sinus; one each for the circumflex and anterior interventricular arteries (or left anterior descending branch) (white arrows) (Figure S5B). In contrast, in the wild‐type heart, there were the two expected ostia, from the right and left aortic sinuses (Figure S5A). In the same heterozygous heart, there were what appeared to be two closing or recently closed muscular VSDs, with the tissue density being less than the rest of the ventricular septum (Figure S6B compared with control in A).

### 3.6 Differential expression of genes essential for cardiogenesis

**FIGURE 7:**
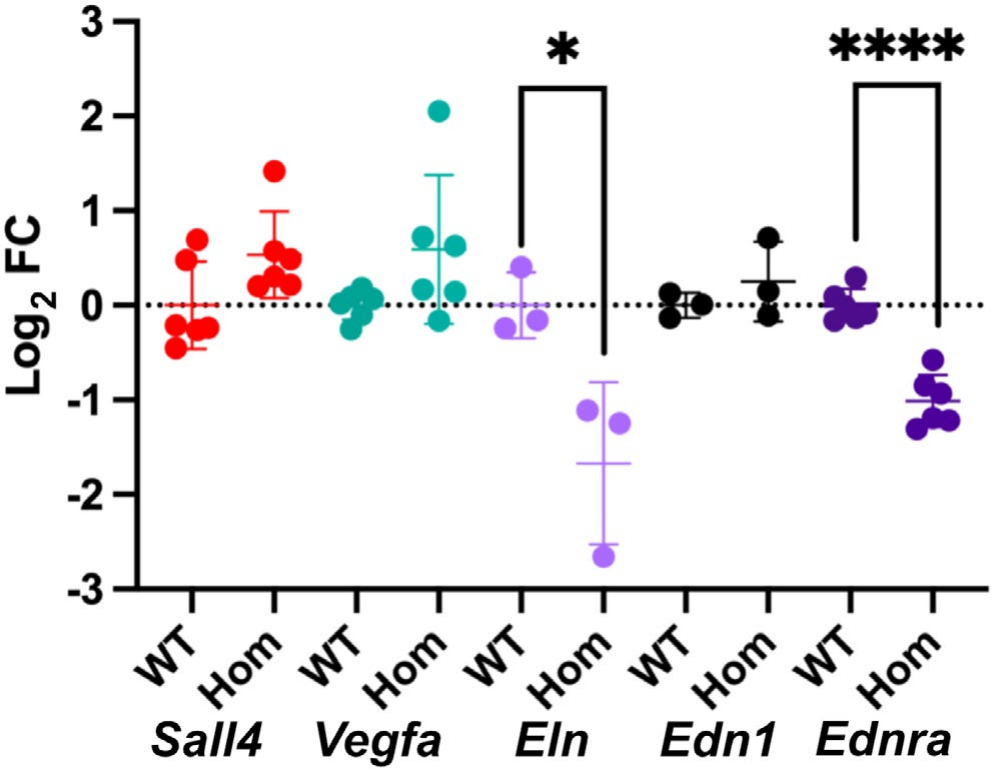
Modulated expression of genes related to cardiogenesis in E12.5 mouse homozygous (*Cdk13* ^*tm1b /tm1b*^) heart. *M*odulated expression of genes related to cardiogenesis in homozygous *Cdk13* ^*tm1b*^ mouse hearts compared to WT was seen, shown as mean log_2_FC ± SD. The difference was statistically significant only in case of *Eln* and *Ednra* (*p* < 0.05, unpaired *t*‐test). *Sall4*, Sal‐like 4; *Vegfa*, Vascular endothelial growth factor A; *Edn1*, Endothelin 1; *Ednra*, Endothelin A receptor; *Eln*, Elastin; SD, standard deviation; FC, fold change. Single asterisk (*) denotes *p* = 0.0352 whereas four asterisks (****) denote *p* < 0.0001.

In order to determine if the *Cdk13* null mutation results in modulated expression of genes related to heart development and CHD, a number of genes were selected for further study. Sal‐like 4 (*Sall4*), vascular endothelial growth factor A (*Vegfa*), endothelin 1 (*Edn1*), endothelin A receptor (*Ednra*) and the extracellular protein elastin (*Eln*) were selected for expression analysis by RT‐qPCR comparing WT (*Cdk13* ^*+/+*^) with homozygous (*Cdk13* ^*tm1b/tm1b*^) hearts at E12.5. Prior to qPCR, expression of these genes of interest at the selected mouse embryonic age (E12.5) was determined from *Bgee* (Bastian et al., 2021) and MGI (Baldarelli et al., 2021), and their interactions with heart development pathways were analysed using *STRING* (Szklarczyk et al., 2015). Significant change in expression was noted only for *Eln* (*p* = 0.0352, unpaired *t*‐test) and *Ednra* (*p* < 0.0001, unpaired *t*‐test) in homozygous (*Cdk13* ^*tm1b/tm1b*^) hearts compared to WT (*Cdk13* ^*+/+*^) (Figure 7).

## 4 DISCUSSION

There have been 91 pathogenic or likely pathogenic variants associated with CDK13‐related disorder to date, affecting at least 172 individuals. Of 138 individuals with a known phenotype, 58 (42%) had a CHD. However, there is variability in expression of the heart phenotype, even in individuals with the same variant. In the study described here, 17% of heterozygous mouse hearts had a CHD.

This is the second published work detailing a *Cdk13* null mutant mouse. The first study was the analysis of the parent allele, *Cdk13* ^*tm1a*^ (Nováková et al., 2019). The *Cdk13* ^*tm1a*^ allele produces a CDK13 protein that is truncated around exon 2, which has been proposed to represent a hypomorphic allele. Certainly, it is known that the *tm1a* allele may not result in a true knockout, and hence can be phenotypically hypomorphic or WT (Ryder et al., 2014). *Cdk13* ^*tm1d*^ mice were then produced, in which exons 3 and 4 were deleted (Nováková et al., 2019). CDK13 protein levels in *Cdk13* ^*tm1d/+*^ embryonic brain tissue was reduced, and absent in *Cdk13* ^*tm1d/tm1d*^ mice. The results of lethality assessments were consistent between this (Table 1) and the previous work by Nováková et al. (2019). This points to a clear and essential role of *CDK13* for successful embryogenesis. Novakova et al. used micro‐CT to try and determine the cause of embryonic lethality (Nováková et al., 2019). They noted that ventricular wall thickness was thinner in *Cdk13* ^*tm1a/tm1a*^ mice compared to control and their results suggested heart failure as the cause of death. Furthermore, ultrasound Doppler imaging found that blood flow was impaired by E15.5. However, they did not perform a focused analysis for the presence of congenital heart disorders.

X‐gal staining shows that *Cdk13* is widely expressed in the developing heart. The low levels of *Cdk13* expression seen in homozygous hearts by qPCR suggests that there is some ‘leakiness’, with the knockout not complete. Although normal levels of mRNA were seen here in heterozygous hearts, the protein levels could be reduced in the heterozygous and absent in homozygous hearts in comparison to controls, as in *Cdk13* ^*tm1d*^ mice with similar deletion (Nováková et al., 2019).

BAV is the most common CHD in humans, occurring in over 2% of the population. BAV is not always symptomatic, but some will develop sequalae such as aortic dilatation and aortic stenosis or regurgitation, and it can be associated with other abnormalities such as aortic coarctation (SHAH ET AL., 2018, LIONCINO ET AL., 2022). heart failure, aortic dissection and sudden death do occur (Bravo‐Jaimes & Prakash, 2020). BAV is seen in both CDK13‐related disorder and three heterozygous mouse embryos in this work, suggesting that it may be a specific CHD phenotype linked with *CDK13*. Six individuals with CDK13‐related disorder had BAV, with three of these harbouring the N1097K variant (Acharya et al., 2021). However, these three individuals had homozygous variants and were from the same consanguineous family. In the study described here, of the 51 *CDK13* ^*TM1B*^ heterozygous and homozygous hearts analysed, three of the heterozygous hearts had a BAV (6%). The two‐sinus type of BAV, seen in *CDK13* ^*TM1B*^ E15.5 heterozygous mutant hearts (also described as bisinuate, bileaflet), had left and right aortic leaflets and sinuses, with the non‐coronary leaflet not discerned. This type of BAV occurs in 5%–7% of cases in humans (Michelena et al., 2021). It has been suggested that lack of the intercalated leaflet swellings (intercalated valve cushions) in development results in the absence of the non‐coronary leaflet (Anderson et al., 2014). SOX17‐PDGRB signalling is known to be involved in the formation of the two‐sinus type (Lu et al., 2022). It is not known if CDK13 is involved in these signalling mechanisms.

One of the heterozygous hearts that had BAV also had an abnormal pulmonary valve (Figure 4B). Instead of the normal three pulmonary valve leaflets (anterior, left and right), there were four leaflets. In contrast to BAV, quadricuspid aortic or pulmonary valve is a rare congenital defect. However, when a quadricuspid pulmonary valve (QPV) is present, it can occur with bicuspid (two‐sinus) aortic valve defects (Davia et al., 1977), as has been seen in the *Cdk13* ^*tm1b*^ heterozygote heart in Figure 4B. However, not all individuals with QPV are symptomatic and it has not been associated with pulmonary valve stenosis or insufficiency (Berdajs et al., 2003).

Two aortic ostia in the respective sinuses were seen in the embryonic hearts analysed by HREM. However, one of P6 hearts had three aortic ostia. The left sinus has two ostia; one of these led to the circumflex artery, with the other to the anterior interventricular artery (or the left anterior descending branch). However, this arrangement could be considered a rare but normal variation (Angelini, 2002).

While static imaging such as HREM does not allow assessment of flow across a valve, there are other indicators of valve dysfunction and discrepant outflow septation. Pulmonary stenosis is likely to be present in three P6 and at least one of the E15.5 *Cdk13* ^*tm1b*^ mutant hearts. The P6 hearts were similar in presentation, with thickened ventricular walls and a narrow right ventricular outflow region into the pulmonary trunk. The E15.5 homozygous heart had a noticeably enlarged proximal aorta and small pulmonary trunk, suggesting that there may have been reduced flow across the valve. As this embryo died before harvesting, we cannot be certain whether these phenotypes are a consequence of the *Cdk13* null mutation or due to growth delay. However, we suggest such a difference in size is not a normal occurrence that would take place in development. Therefore, we propose this small pulmonary trunk and large aorta is a true phenotype as a consequence of this *Cdk13* deletion, resulting in pulmonary stenosis.

It is notable that 17% of heterozygous E15.5 hearts had CHD. Other features included poor growth and pericardial effusion. Pericardial effusion in the embryo has been used as an indicator of cardiac failure and impending demise (Shen et al., 2005; Yu et al., 2004) and was seen in 4% of E15.5 embryos, suggesting poor cardiac function. Abnormal trabeculae (hypertrophic, sponge‐like appearance) were seen in the *Cdk13* ^*tm1b*^ mutant hearts, and this has been seen in other pathological conditions, such as adaptation to increased pressure on the right ventricle (Fatemifar et al., 2019; Loukas et al., 2013; van de Veerdonk et al., 2014). However, abnormalities with trabeculation may not always present pathologically (Petersen et al., 2023). The clinical significance of this is therefore unclear. Three out of four homozygous hearts had a cobblestone appearance, which could be due to post‐mortem embryonic resorption as these embryos died before harvesting. Therefore, it is uncertain if the phenotypes observed could partially be due to the embryo being at an earlier developmental stage.

The uncertainty surrounding how *CDK13* variants cause the features of CDK13‐related disorder makes it difficult to adequately counsel families with other variants affecting CDK13, such as intragenic deletions and duplications. It is interesting that the mouse model suggests that homozygous loss of function of CDK13 results in embryonic lethality, and yet a family has been reported with homozygous variants in CDK13 (Acharya et al., 2021). Of note is that the phenotype reported in the consanguineous family is that of Wolfram Syndrome (diabetes insipidus and diabetes mellitus with optical atrophy, and deafness, DIDMOAD) (Pallotta et al., 2019), which is quite distinct from the phenotype of CDK13‐related disorder described so far. Optic atrophy was not present in the reported siblings and the only real similarity was BAV and clinodactyly. These children had no intellectual disability or facial dysmorphism (Acharya et al., 2021), which is present in CDK13‐related disorder. Whilst it is possible that the onset of diabetes and deafness might have not yet developed in the young individuals with CDK13‐related disorder, it is perhaps more likely that this is a distinct syndrome because of a different mechanism of action. The variant reported in this family (p.Asn1097Lys) is outside of the kinase domain and was predicted to cause a hypomorphic allele (Acharya et al., 2021).

Several mechanisms have been suggested in CDK13‐related disorder. Gain of function has been proposed due to the clustering of missense variants within the kinase domain, as well as a dominant negative effect due to sequestration of cyclin K (Hamilton & Suri, 2019; Sifrim et al., 2016). However, loss of function or haploinsufficiency is also possible (Gibbs et al., 2023).

Little is known of downstream targets for CDK13, but the range of phenotypes seen in humans indicates that it is involved in a variety of signalling pathways, and hence, pathogenic variants have a widespread effect. A recent study investigated the molecular pathways associated with craniofacial defects resulting from CDK13 deletion (Hampl et al., 2024).

Therefore, to provide a greater understanding of potential downstream targets of *Cdk13*, several genes were selected, due to their critical but differing roles in cardiogenesis, to be analysed by qPCR to see if they showed differential expression in the *Cdk13* ^*tm1b*^ E12.5 homozygous hearts in comparison to WT controls. Significant differential expression was not seen for the zinc finger transcription factor *Sall4*, which has been associated with ASDs, VSDs and conduction anomalies (Kohlhase, 1993), or the angiogenic growth factor *Vegfa* which has been associated with AVSD (Ackerman et al., 2012; Redig et al., 2014). Differential expression was also not significant for the endothelin 1 (*Edn1*) gene which is associated with cardiac neural crest cells (CNCC) (Kurihara et al., 1999), and their defective migration is associated with CHDs (Thattaliyath & Firulli, 2024). Conversely, two genes were downregulated in the *Cdk13* ^*tm1b/tm1b*^ E12.5 heart, compared to controls. Endothelin A receptor (*Ednra*) is the receptor with the highest affinity for *Edn1*. *Ednra* is essential for correct cardiac neural crest cell (CNCC) migration (Fritz et al., 2019). Mice deficient in *Ednra* have heart defects such as perimembranous VSD, DORV and transposition of the great arteries (Clouthier et al., 1998). The decreased expression in *Ednra* observed in the *Cdk13* ^*tm1b*^ homozygous heart is therefore suggestive of CNCC being affected in individuals with a *Cdk13* variant. Another gene that was decreased was elastin (*Eln*). *Eln* encodes an extracellular matrix protein ELN, that provides elasticity to the arterial wall, allowing both stretch and recoil with each cardiac cycle (Lin et al., 2022). Williams syndrome is a rare microdeletion disorder which is strongly associated with *ELN* deletion (Ewart et al., 1993; Pérez Jurado et al., 1996). *ELN* mutations are commonly associated with aortic stenosis, predominately supravalvar, but other defects associated with it include tetralogy of Fallot, ASD, PS, BAV and VSD (Kozel et al., 2021). Deletion mutation of *ELN* is possibly associated with growth abnormalities (Kozel et al., 2021), which was seen in *Cdk13* ^*tm1b*^ homozygous mice with significantly reduced CRL (*p* < 0.05) at both E12.5 and E15.5. These defects are consistent with the abnormalities seen in individuals with CDK13‐related disorder and herein in the *Cdk13* ^*tm1b*^ mutant mouse. These two genes are of interest and worthy of further study with regard to *Cdk13* function and heart morphogenesis.

The types of defects observed in the heterozygous mouse hearts are consistent with the human phenotype, where VSDs, DORV and BAV were also present. However, the specific type of VSD identified in individuals with CDK13‐related disorder was unfortunately not reported in the literature. It can be challenging to gather detailed clinical information in rare diseases such as CDK13‐related disorder, when affected individuals are spread across multiple medical centres worldwide. Closer collaboration between clinicians and researchers and consistent use of standardised phenotype classification systems would go some way to improving this situation. Unfortunately, this is not straightforward due to the use of multiple classification systems, such as Human Phenotype Ontology terms, European Paediatric Cardiac Codes and ICD11, with each system having its own limitations. The presence or absence of CHD in CDK13‐related disorder does not appear to show a specific genotype/phenotype correlation. The finding of reduced penetrance of CHD in the heterozygous mice studied here demonstrates an important role of CDK13 in heart morphogenesis but implies that there may be additional modifying factors.

While it is likely that the full phenotypic spectrum of CDK13‐related disorder has not yet been established, we have provided further evidence of the role of *CDK13* in cardiogenesis. This supports CHD as a significant part of the phenotype, even if CHD does not occur in every single individual with CDK13‐related disorder. Like many other rare genetic conditions (Jongmans et al., 2006; Li et al., 1997; Roberts et al., 2013), expressivity and penetrance of specific abnormalities such as CHD is variable. This work demonstrates that loss of function of CDK13 is associated with CHD in mice, even in the heterozygous state, indicating haploinsufficiency of *Cdk13* results in CHD. This analysis and our previous study (Waheed‐Ullah et al., 2024) demonstrate the utility of HREM for detailed morphological analysis in the mouse embryonic heart, providing enough resolution to classify structural defects such as BAV, and to assess finer details such as the endocardial cushions, valve leaflets, myocardial trabeculae, small muscular VSDs and to classify the types of VSD. Understanding the effects of reduced function of CDK13 in the mouse heart, and being able to compare the CHDs seen here, with that of individuals with CDK13‐related disorder, allows us to strengthen the phenotypic associations and potentially understand the mechanism behind this condition. Going forward, this should enable us to refine and improve care for affected individuals and their families.

## AUTHOR CONTRIBUTIONS

JDB and SL conceived the idea and designed the experiments, whereas QWU, AW, SR and AAS performed the experiments. QWU, FB, AW, AA, SR, JDB and SL performed data analysis. MPH provided additional intellectual input during the project. SL and AA wrote the first draft. All authors reviewed and edited the manuscript and consented to the submission of the final draft.

## FUNDING INFORMATION

We would also like to express our appreciation to the Higher Education Department, KPK, Pakistan, who have funded QWU, the BHF for funding AW a British Heart Foundation Clinical Research Training Fellowship (FS/14/51/30879), The Hashemite University, Jordan for funding AA and Applied Science Private University, Jordan for funding AAS.

## CONFLICT OF INTEREST STATEMENT

The authors declare they have no conflicts of interest.

## Supporting information


**Data S1:** Supplementary Information.

## Data Availability

Data supporting the HREM findings of this study are available from the corresponding author upon reasonable request.

## References

[joa14175-bib-0001] Acharya, A. , Raza, S.I. , Anwar, M.Z. , Bharadwaj, T. , Liaqat, K. , Khokhar, M.A.S. et al. (2021) Wolfram‐like syndrome with bicuspid aortic valve due to a homozygous missense variant in CDK13. Journal of Human Genetics, 66, 1009–1018. 33879837 10.1038/s10038-021-00922-0 PMC8472924

[joa14175-bib-0002] Ackerman, C. , Locke, A.E. , Feingold, E. , Reshey, B. , Espana, K. , Thusberg, J. et al. (2012) An excess of deleterious variants in VEGF‐A pathway genes in Down‐syndrome‐associated atrioventricular septal defects. American Journal of Human Genetics, 91, 646–659. 23040494 10.1016/j.ajhg.2012.08.017 PMC3484504

[joa14175-bib-0003] Anderson, R.H. , Mohun, T.J. , Spicer, D.E. , Bamforth, S.D. , Brown, N.A. , Chaudhry, B. et al. (2014) Myths and realities relating to development of the arterial valves. Journal of Cardiovascular Development and Disease, 1, 177–200.

[joa14175-bib-0004] Anderson, R.H. , Wessels, A. & Vettukattil, J.J. (2010) Morphology and morphogenesis of atrioventricular septal defect with common atrioventricular junction. World J Pediatr Congenit Heart Surg, 1, 59–67. 23804724 10.1177/2150135109360813

[joa14175-bib-0005] Angelini, P. (2002) Coronary artery anomalies—current clinical issues: definitions, classification, incidence, clinical relevance, and treatment guidelines. Texas Heart Institute Journal, 29, 271–278. 12484611 PMC140289

[joa14175-bib-0006] Baldarelli, R.M. , Smith, C.M. , Finger, J.H. , Hayamizu, T.F. , McCright, I.J. , Xu, J. et al. (2021) The mouse gene expression database (GXD): 2021 update. Nucleic Acids Research, 49, D924–D931. 33104772 10.1093/nar/gkaa914 PMC7778941

[joa14175-bib-0007] Bastian, F.B. , Roux, J. , Niknejad, A. , Comte, A. , Fonseca Costa, S.S. , de Farias, T.M. et al. (2021) The Bgee suite: integrated curated expression atlas and comparative transcriptomics in animals. Nucleic Acids Research, 49, D831–d847. 33037820 10.1093/nar/gkaa793 PMC7778977

[joa14175-bib-0008] Berdajs, D. , Lajos, P. , Zünd, G. & Turina, M. (2003) The quadricuspid pulmonary valve: its importance in the Ross procedure. The Journal of Thoracic and Cardiovascular Surgery, 125, 198–199. 12539007 10.1067/mtc.2003.115

[joa14175-bib-0009] Blanco, M.J. , Learte, A.I.R. , Marchena, M.A. , Muñoz‐Sáez, E. , Cid, M.A. , Rodríguez‐Martín, I. et al. (2018) Tracing gene expression through detection of β‐galactosidase activity in whole mouse embryos. Journal of Visualized Experiments (136), 57785. 30010638 10.3791/57785 PMC6101999

[joa14175-bib-0075] Bostwick, B.L. , McLean, S. , Posey, J.E. , Streff, H.E. , Gripp, K.W. , Blesson, A. et al. (2017) Phenotypic and molecular characterisation of CDK13‐related congenital heart defects, dysmorphic facial features and intellectual developmental disorders. Genome Medicine, 9(1), 73. Available from: 10.1186/s13073-017-0463-8 28807008 PMC5557075

[joa14175-bib-0010] Bravo‐Jaimes, K. & Prakash, S.K. (2020) Genetics in bicuspid aortic valve disease: where are we? Progress in Cardiovascular Diseases, 63, 398–406. 32599026 10.1016/j.pcad.2020.06.005 PMC7530017

[joa14175-bib-0011] Burns, T. , Yang, Y. , Hiriart, E. & Wessels, A. (2016) The dorsal mesenchymal protrusion and the pathogenesis of atrioventricular septal defects. J Cardiovasc Dev Dis, 3(4), 29. 28133602 10.3390/jcdd3040029 PMC5267359

[joa14175-bib-0081] Carneiro, T.N. , Krepischi, A.C. , Costa, S.S. , Tojal da Silva, I. , Vianna‐Morgante, A.M. , Valieris, R. et al. (2018) Utility of trio‐based exome sequencing in the elucidation of the genetic basis of isolated syndromic intellectual disability: illustrative cases. The Application of Clinical Genetics, 11, 93–98. Available from: 10.2147/tacg.S165799 30174453 PMC6110279

[joa14175-bib-0012] Chepelev, I. (2012) Detection of RNA editing events in human cells using high‐throughput sequencing. Methods in Molecular Biology, 815, 91–102. 22130986 10.1007/978-1-61779-424-7_8 PMC4184133

[joa14175-bib-0013] Clouthier, D.E. , Hosoda, K. , Richardson, J.A. , Clay Williams, S. , Yanagisawa, H. , Kuwaki, T. et al. (1998) Cranial and cardiac neural crest defects in endothelin‐a receptor‐deficient mice. Development, 125, 813–824. 9449664 10.1242/dev.125.5.813

[joa14175-bib-0082] Cui, X. , Wu, X. , Wang, H. , Zhang, S. , Wang, W. & Jing, X. (2022a) Genetic of preimplantation diagnosis of dysmorphic facial features and intellectual developmental disorder (CHDFIDD) without congenital heart defects. Molecular Genetics & Genomic Medicine, 10(2), e1863. Available from: 10.1002/mgg3.1863 35034425 PMC8830809

[joa14175-bib-0083] Cui, D. , Wang, S. , Zhang, A. , Liu, A. & Hu, Q. (2022b) Case report: hemophagocytic lymphohistiocytosis prior to the onset of leukemia in a boy with CDK13‐related disorder. Frontiers in Genetics, 13, 858668. Available from: 10.3389/fgene.2022.858668 35651941 PMC9149378

[joa14175-bib-0014] Davia, J.E. , Fenoglio, J.J. , Decastro, C.M. , Mcallister, H.A., Jr. & Cheitlin, M.D. (1977) Quadricuspid semilunar valves. Chest, 72, 186–189. 884980 10.1378/chest.72.2.186

[joa14175-bib-0015] Dong, X. , Chen, G. , Cai, Z. , Li, Z. , Qiu, L. , Xu, H. et al. (2018) CDK13 RNA over‐editing mediated by ADAR1 associates with poor prognosis of hepatocellular carcinoma patients. Cellular Physiology and Biochemistry, 47, 2602–2612. 29996118 10.1159/000491656

[joa14175-bib-0016] Even, Y. , Escande, M.L. , Fayet, C. & Genevière, A.M. (2016) CDK13, a kinase involved in pre‐mRNA splicing, is a component of the Perinucleolar compartment. PLoS One, 11, e0149184. 26886422 10.1371/journal.pone.0149184 PMC4757566

[joa14175-bib-0017] Ewart, A.K. , Morris, C.A. , Atkinson, D. , Jin, W. , Sternes, K. , Spallone, P. et al. (1993) Hemizygosity at the elastin locus in a developmental disorder, Williams syndrome. Nature Genetics, 5, 11–16. 7693128 10.1038/ng0993-11

[joa14175-bib-0018] Fatemifar, F. , Feldman, M.D. , Oglesby, M. & Han, H.C. (2019) Comparison of biomechanical properties and microstructure of trabeculae Carneae, papillary muscles, and myocardium in the human heart. Journal of Biomechanical Engineering, 141, 210071–2100710. 10.1115/1.4041966 PMC6298537 30418486

[joa14175-bib-0085] Firth, H.V. , Richards, S.M. , Bevan, A.P. , Clayton, S. , Corpas, M. , Rajan, D. et al. (2009) DECIPHER: database of chromosomal imbalance and phenotype in humans using ensembl resources. American Journal of Human Genetics, 84(4), 524–533. Available from: 10.1016/j.ajhg.2009.03.010 19344873 PMC2667985

[joa14175-bib-0019] Franklin, R.C.G. , Béland, M.J. , Colan, S.D. , Walters, H.L., III , Aiello, V.D. , Anderson, R.H. et al. (2017) Nomenclature for congenital and paediatric cardiac disease: the international Paediatric and congenital cardiac code (IPCCC) and the eleventh iteration of the international classification of diseases (ICD‐11). Cardiology in the Young, 27, 1872–1938. 29286277 10.1017/S1047951117002244

[joa14175-bib-0020] Fritz, K.R. , Zhang, Y. & Ruest, L.B. (2019) Cdc42 activation by endothelin regulates neural crest cell migration in the cardiac outflow tract. Developmental Dynamics, 248, 795–812. 31219639 10.1002/dvdy.75

[joa14175-bib-0021] Geyer, S.H. , Reissig, L. , Rose, J. , Wilson, R. , Prin, F. , Szumska, D. et al. (2017) A staging system for correct phenotype interpretation of mouse embryos harvested on embryonic day 14 (E14.5). Journal of Anatomy, 230, 710–719. 28185240 10.1111/joa.12590 PMC5382591

[joa14175-bib-0022] Gibbs, M. , Poulin, A. , Xi, Y. & Hashemi, B. (2023) A prenatal presentation of CDK13‐related disorder with a novel pathogenic variant. Case Reports in Genetics, 2023, 3437706. 37351084 10.1155/2023/3437706 PMC10284631

[joa14175-bib-0023] Gierut, J.J. , Jacks, T.E. & Haigis, K.M. (2014) Whole‐mount X‐gal staining of mouse tissues. Cold Spring Harbor Protocols, 2014, 417–419. 24692489 10.1101/pdb.prot073452 PMC4169236

[joa14175-bib-0073] Hamilton, M.J. , Caswell, R.C. , Canham, N. , Cole, T. , Firth, H.V. , Foulds, N. et al. (2018) Heterozygous mutations affecting the protein kinase domain of CDK13 cause a syndromic form of developmental delay and intellectual disability. Journal of Medical Genetics, 55(1), 28–38. Available from: 10.1136/jmedgenet-2017-104620 29021403 PMC5749303

[joa14175-bib-0024] Hamilton, M.J. & Suri, M. (2019) CDK13‐related disorder. Advances in Genetics, 103, 163–182. 30904094 10.1016/bs.adgen.2018.11.001

[joa14175-bib-0025] Hampl, M. , Jandová, N. , Lusková, D. , Nováková, M. , Szotkowská, T. , Čada, Š. et al. (2024) Early embryogenesis in CHDFIDD mouse model reveals facial clefts and altered cranial neurogenesis. Disease Models & Mechanisms, 17(6), dmm050261. 38511331 10.1242/dmm.050261 PMC11212636

[joa14175-bib-0026] Hellemans, J. , Mortier, G. , De Paepe, A. , Speleman, F. & Vandesompele, J. (2007) qBase relative quantification framework and software for management and automated analysis of real‐time quantitative PCR data. Genome Biology, 8, R19. 17291332 10.1186/gb-2007-8-2-r19 PMC1852402

[joa14175-bib-0027] Homsy, J. , Zaidi, S. , Shen, Y. , Ware, J.S. , Samocha, K.E. , Karczewski, K.J. et al. (2015) De novo mutations in congenital heart disease with neurodevelopmental and other congenital anomalies. Science, 350, 1262–1266. 26785492 10.1126/science.aac9396 PMC4890146

[joa14175-bib-0028] Jin, S.C. , Homsy, J. , Zaidi, S. , Lu, Q. , Morton, S. , DePalma, S.R. et al. (2017) Contribution of rare inherited and de novo variants in 2,871 congenital heart disease probands. Nature Genetics, 49, 1593–1601. 28991257 10.1038/ng.3970 PMC5675000

[joa14175-bib-0029] Jongmans, M.C. , Admiraal, R.J. , Van Der Donk, K.P. , Vissers, L.E. , Baas, A.F. , Kapusta, L. et al. (2006) CHARGE syndrome: the phenotypic spectrum of mutations in the CHD7 gene. Journal of Medical Genetics, 43, 306–314. 16155193 10.1136/jmg.2005.036061 PMC2563221

[joa14175-bib-0030] Kacpura, H. & Rodriguez‐Buritica, D. (2021) eP226—Septo‐optic dysplasia in a patient with CDK13‐related disorder. Molecular Genetics and Metabolism, 132, S145.

[joa14175-bib-0031] Kaufman, M.H. (1995) The atlas of mouse development. London and San Diego: Academic Press.

[joa14175-bib-0032] Kim, H.E. , Kim, D.G. , Lee, K.J. , Son, J.G. , Song, M.Y. , Park, Y.M. et al. (2012) Frequent amplification of CENPF, GMNN and CDK13 genes in hepatocellular carcinomas. PLoS One, 7, e43223. 22912832 10.1371/journal.pone.0043223 PMC3418236

[joa14175-bib-0033] Kohlhase, J. (1993) In: Adam, M.P. , Feldman, J. , Mirzaa, G.M. , Pagon, R.A. , Wallace, S.E. , Bean, L.J.H. et al. (Eds.) *GeneReviews*(*®*)SALL4‐related disorders Seattle (WA): University of Washington. 20301547

[joa14175-bib-0034] Koressaar, T. & Remm, M. (2007) Enhancements and modifications of primer design program Primer3. Bioinformatics, 23, 1289–1291. 17379693 10.1093/bioinformatics/btm091

[joa14175-bib-0035] Kozel, B.A. , Barak, B. , Kim, C.A. , Mervis, C.B. , Osborne, L.R. , Porter, M. et al. (2021) Williams syndrome Nature Reviews. Disease Primers, 7, 42. 10.1038/s41572-021-00276-z PMC9437774 34140529

[joa14175-bib-0036] Kurihara, H. , Kurihara, Y. , Nagai, R. & Yazaki, Y. (1999) Endothelin and neural crest development. Cellular and Molecular Biology (Noisy‐le‐Grand, France), 45, 639–651. 10512195

[joa14175-bib-0084] Landrum, M.J. , Lee, J.M. , Benson, M. , Brown, G.R. , Chao, C. , Chitipiralla, S. et al. (2018) ClinVar: improving access to variant interpretations and supporting evidence. Nucleic Acids Research, 46(D1), D1062–d7. Available from: 10.1093/nar/gkx1153 29165669 PMC5753237

[joa14175-bib-0037] Li, Q.Y. , Newbury‐Ecob, R.A. , Terrett, J.A. , Wilson, D.I. , Curtis, A.R. , Yi, C.H. et al. (1997) Holt‐Oram syndrome is caused by mutations in TBX5, a member of the Brachyury (T) gene family. Nature Genetics, 15, 21–29. 8988164 10.1038/ng0197-21

[joa14175-bib-0038] Liang, K. , Gao, X. , Gilmore, J.M. , Florens, L. , Washburn, M.P. , Smith, E. et al. (2015) Characterization of human cyclin‐dependent kinase 12 (CDK12) and CDK13 complexes in C‐terminal domain phosphorylation, gene transcription, and RNA processing. Molecular and Cellular Biology, 35, 928–938. 25561469 10.1128/MCB.01426-14 PMC4333096

[joa14175-bib-0039] Lin, C.J. , Cocciolone, A.J. & Wagenseil, J.E. (2022) Elastin, arterial mechanics, and stenosis. American Journal of Physiology. Cell Physiology, 322, C875–c886. 35196168 10.1152/ajpcell.00448.2021 PMC9037699

[joa14175-bib-0040] Lioncino, M. , Monda, E. , Verrillo, F. , Moscarella, E. , Calcagni, G. , Drago, F. et al. (2022) Hypertrophic cardiomyopathy in RASopathies: diagnosis, clinical characteristics, prognostic implications, and management. Heart Failure Clinics, 18, 19–29. 34776080 10.1016/j.hfc.2021.07.004 PMC9674037

[joa14175-bib-0041] Loughna, S. & Henderson, D. (2007) Methodologies for staining and visualisation of beta‐galactosidase in mouse embryos and tissues. Methods in Molecular Biology, 411, 1–11. 18287634 10.1007/978-1-59745-549-7_1

[joa14175-bib-0042] Loukas, M. , Housman, B. , Blaak, C. , Kralovic, S. , Tubbs, R.S. & Anderson, R.H. (2013) Double‐chambered right ventricle: a review. Cardiovascular Pathology, 22, 417–423. 23701985 10.1016/j.carpath.2013.03.004

[joa14175-bib-0043] Lu, P. , Wang, P. , Wu, B. , Wang, Y. , Liu, Y. , Cheng, W. et al. (2022) A SOX17‐PDGFB signaling axis regulates aortic root development. Nature Communications, 13, 4065. 10.1038/s41467-022-31815-1 PMC9279414 35831318

[joa14175-bib-0044] Michelena, H.I. , Della Corte, A. , Evangelista, A. , Maleszewski, J.J. , Edwards, W.D. , Roman, M.J. et al. (2021) International consensus statement on nomenclature and classification of the congenital bicuspid aortic valve and its aortopathy, for clinical, surgical, interventional and research purposes. The Journal of Thoracic and Cardiovascular Surgery, 162, e383–e414. 34304896 10.1016/j.jtcvs.2021.06.019

[joa14175-bib-0079] Morison, L.D. , van Reyk, O. , Forbes, E. , Rouxel, F. , Faivre, L. , Bruinsma, F. et al. (2023) CDK13‐related disorder: a deep characterization of speech and language abilities and addition of 33 novel cases. European Journal of Human Genetics, 31(7), 793–804. Available from: 10.1038/s41431-022-01275-8 36599938 PMC10325997

[joa14175-bib-0045] Mu, J. , Slevin, J.C. , Qu, D. , Mccormick, S. & Adamson, S.L. (2008) In vivo quantification of embryonic and placental growth during gestation in mice using micro‐ultrasound. Reproductive Biology and Endocrinology, 6, 34. 18700008 10.1186/1477-7827-6-34 PMC2527569

[joa14175-bib-0046] Nováková, M. , Hampl, M. , Vrábel, D. , Procházka, J. , Petrezselyová, S. , Procházková, M. et al. (2019) Mouse model of congenital heart defects, dysmorphic facial features and intellectual developmental disorders as a result of non‐functional CDK13. Frontiers in Cell and Development Biology, 7, 155. 10.3389/fcell.2019.00155 PMC6694211 31440507

[joa14175-bib-0047] Ossa Galvis, M.M. , Bhakta, R.T. , Tarmahomed, A. & Mendez, M.D. (2023) Cyanotic heart disease. Treasure Island (FL): StatPearls Publishing LLC. 29763177

[joa14175-bib-0048] Pallotta, M.T. , Tascini, G. , Crispoldi, R. , Orabona, C. , Mondanelli, G. , Grohmann, U. et al. (2019) Wolfram syndrome, a rare neurodegenerative disease: from pathogenesis to future treatment perspectives. Journal of Translational Medicine, 17, 238. 31337416 10.1186/s12967-019-1993-1 PMC6651977

[joa14175-bib-0049] Pérez Jurado, L.A. , Peoples, R. , Kaplan, P. , Hamel, B.C. & Francke, U. (1996) Molecular definition of the chromosome 7 deletion in Williams syndrome and parent‐of‐origin effects on growth. American Journal of Human Genetics, 59, 781–792. 8808592 PMC1914804

[joa14175-bib-0050] Petersen, S.E. , Jensen, B. , Aung, N. , Friedrich, M.G. , McMahon, C.J. , Mohiddin, S.A. et al. (2023) Excessive Trabeculation of the left ventricle. In: Excessive Trabeculation of the left ventricle: JACC: cardiovascular imaging expert panel paper, Vol. 16. JACC Cardiovasc Imaging, pp. 408–425. 10.1016/j.jcmg.2022.12.026 PMC9988693 36764891

[joa14175-bib-0051] Redig, J.K. , Fouad, G.T. , Babcock, D. , Reshey, B. , Feingold, E. , Reeves, R.H. et al. (2014) Allelic interaction between CRELD1 and VEGFA in the pathogenesis of cardiac atrioventricular septal defects. AIMS Genet, 1, 1–19. 25328912 10.3934/genet.2014.1.1#sthash.jksuJTeC.dpuf PMC4200510

[joa14175-bib-0052] Richter, F. , Morton, S.U. , Kim, S.W. , Kitaygorodsky, A. , Wasson, L.K. , Chen, K.M. et al. (2020) Genomic analyses implicate noncoding de novo variants in congenital heart disease. Nature Genetics, 52, 769–777. 32601476 10.1038/s41588-020-0652-z PMC7415662

[joa14175-bib-0053] Roberts, A.E. , Allanson, J.E. , Tartaglia, M. & Gelb, B.D. (2013) Noonan syndrome. Lancet, 381, 333–342. 23312968 10.1016/S0140-6736(12)61023-X PMC4267483

[joa14175-bib-0080] Rouxel, F. , Relator, R. , Kerkhof, J. , McConkey, H. , Levy, M. , Dias, P. et al. (2022) CDK13‐related disorder: report of a series of 18 previously unpublished individuals and description of an epigenetic signature. Genetics in Medicine. Available from: 10.1016/j.gim.2021.12.016 35063350

[joa14175-bib-0054] Ruiz‐Villalba, A. , Mattiotti, A. , Gunst, Q.D. , Cano‐Ballesteros, S. , van den Hoff, M.J.B. & Ruijter, J.M. (2017) Reference genes for gene expression studies in the mouse heart. Scientific Reports, 7, 24. 28154421 10.1038/s41598-017-00043-9 PMC5428317

[joa14175-bib-0055] Ryder, E. , Doe, B. , Gleeson, D. , Houghton, R. , Dalvi, P. , Grau, E. et al. (2014) Rapid conversion of EUCOMM/KOMP‐CSD alleles in mouse embryos using a cell‐permeable Cre recombinase. Transgenic Research, 23, 177–185. 24197666 10.1007/s11248-013-9764-x PMC3890051

[joa14175-bib-0056] Shah, S.Y. , Higgins, A. & Desai, M.Y. (2018) Bicuspid aortic valve: basics and beyond. Cleveland Clinic Journal of Medicine, 85, 779–784. 30289756 10.3949/ccjm.85a.17069

[joa14175-bib-0057] Shen, Y. , Leatherbury, L. , Rosenthal, J. , Yu, Q. , Pappas, M.A. , Wessels, A. et al. (2005) Cardiovascular phenotyping of fetal mice by noninvasive high‐frequency ultrasound facilitates recovery of ENU‐induced mutations causing congenital cardiac and extracardiac defects. Physiological Genomics, 24, 23–36. 16174781 10.1152/physiolgenomics.00129.2005

[joa14175-bib-0058] Sifrim, A. , Hitz, M.P. , Wilsdon, A. , Breckpot, J. , Turki, S.H. , Thienpont, B. et al. (2016) Distinct genetic architectures for syndromic and nonsyndromic congenital heart defects identified by exome sequencing. Nature Genetics, 48, 1060–1065. 27479907 10.1038/ng.3627 PMC5988037

[joa14175-bib-0059] Szklarczyk, D. , Franceschini, A. , Wyder, S. , Forslund, K. , Heller, D. , Huerta‐Cepas, J. et al. (2015) STRING v10: protein‐protein interaction networks, integrated over the tree of life. Nucleic Acids Research, 43, D447–D452. 25352553 10.1093/nar/gku1003 PMC4383874

[joa14175-bib-0060] Taqatqa, A.S. & Vettukattil, J.J. (2021) Atrioventricular septal defects: pathology, imaging, and treatment options. Current Cardiology Reports, 23, 93. 34196822 10.1007/s11886-021-01523-1

[joa14175-bib-0061] Thattaliyath, B.D. & Firulli, A.B. (2024) Neural Crest. Advances in Experimental Medicine and Biology, 1441, 125–143. 38884708 10.1007/978-3-031-44087-8_6

[joa14175-bib-0074] Trinh, J. , Kandaswamy, K.K. , Werber, M. , Weiss, M.E.R. , Oprea, G. , Kishore, S. et al. (2019) Novel pathogenic variants and multiple molecular diagnoses in neurodevelopmental disorders. Journal of Neurodevelopmental Disorders, 11(1), 11. Available from: 10.1186/s11689-019-9270-4 31238879 PMC6593513

[joa14175-bib-0076] Uehara, T. , Takenouchi, T. , Kosaki, R. , Kurosawa, K. , Mizuno, S. & Kosaki, K. (2018) Redefining the phenotypic spectrum of de novo heterozygous CDK13 variants: Three patients without cardiac defects. European Journal of Medical Genetics, 61(5), 243–247. Available from: 10.1016/j.ejmg.2017.12.004 29222009

[joa14175-bib-0062] Untergasser, A. , Cutcutache, I. , Koressaar, T. , Ye, J. , Faircloth, B.C. , Remm, M. et al. (2012) Primer3—new capabilities and interfaces. Nucleic Acids Research, 40, e115. 22730293 10.1093/nar/gks596 PMC3424584

[joa14175-bib-0063] Van De Veerdonk, M.C. , Dusoswa, S.A. , Marcus, J.T. , Bogaard, H.J. , Spruijt, O. , Kind, T. et al. (2014) The importance of trabecular hypertrophy in right ventricular adaptation to chronic pressure overload. The International Journal of Cardiovascular Imaging, 30, 357–365. 24306052 10.1007/s10554-013-0338-z

[joa14175-bib-0077] van den Akker, W.M.R. , Brummelman, I. , Martis, L.M. , Timmermans, R.N. , Pfundt, R. , Kleefstra, T. et al. (2018) De novo variants in CDK13 associated with syndromic ID/DD: Molecular and clinical delineation of 15 individuals and a further review. Clinical Genetics, 93(5), 1000–1007. Available from: 10.1111/cge.13225 29393965

[joa14175-bib-0064] Vandesompele, J. , De Preter, K. , Pattyn, F. , Poppe, B. , Van Roy, N. , De Paepe, A. et al. (2002) Accurate normalization of real‐time quantitative RT‐PCR data by geometric averaging of multiple internal control genes. Genome Biology, 3, Research0034. 12184808 10.1186/gb-2002-3-7-research0034 PMC126239

[joa14175-bib-0065] Waheed‐Ullah, Q. , Wilsdon, A. , Abbad, A. , Rochette, S. , Bu'Lock, F. , Hitz, M.P. et al. (2024) Effect of deletion of the protein kinase PRKD1 on development of the mouse embryonic heart. Journal of Anatomy, 245(1), 70–83. 38419169 10.1111/joa.14033 PMC11161829

[joa14175-bib-0066] Wang, T. , Hoekzema, K. , Vecchio, D. , Wu, H. , Sulovari, A. , Coe, B.P. et al. (2020) Large‐scale targeted sequencing identifies risk genes for neurodevelopmental disorders. Nature Communications, 11, 4932. 10.1038/s41467-020-18723-y PMC7530681 33004838

[joa14175-bib-0067] Webb, S. , Brown, N.A. & Anderson, R.H. (1998) Formation of the atrioventricular septal structures in the normal mouse. Circulation Research, 82, 645–656. 9546373 10.1161/01.res.82.6.645

[joa14175-bib-0068] Weninger, W.J. , Maurer‐Gesek, B. , Reissig, L.F. , Prin, F. , Wilson, R. , Galli, A. et al. (2018) Visualising the cardiovascular system of embryos of biomedical model organisms with high resolution Episcopic microscopy (HREM). Journal of Cardiovascular Development and Disease, 5, 58. 30558275 10.3390/jcdd5040058 PMC6306920

[joa14175-bib-0078] Yakubov, R. , Ayman, A. , Kremer, A.K. & van den Akker, M. (2019) One‐month‐old girl presenting with pseudohypoaldosteronism leading to the diagnosis of CDK13‐related disorder: a case report and review of the literature. Journal of Medical Case Reports, 13(1), 386. Available from: 10.1186/s13256-019-2319-x 31883531 PMC6935476

[joa14175-bib-0069] Ye, J. , Coulouris, G. , Zaretskaya, I. , Cutcutache, I. , Rozen, S. & Madden, T.L. (2012) Primer‐BLAST: a tool to design target‐specific primers for polymerase chain reaction. BMC Bioinformatics, 13, 134. 22708584 10.1186/1471-2105-13-134 PMC3412702

[joa14175-bib-0070] Yim, D. , Dragulescu, A. , Ide, H. , Seed, M. , Grosse‐Wortmann, L. , van Arsdell, G. et al. (2018) Essential modifiers of double outlet right ventricle: revisit with endocardial surface images and 3‐dimensional print models. Circulation. Cardiovascular Imaging, 11, e006891. 29855425 10.1161/CIRCIMAGING.117.006891

[joa14175-bib-0071] Yu, Q. , Shen, Y. , Chatterjee, B. , Siegfried, B.H. , Leatherbury, L. , Rosenthal, J. et al. (2004) ENU induced mutations causing congenital cardiovascular anomalies. Development, 131, 6211–6223. 15548583 10.1242/dev.01543

[joa14175-bib-0072] Zaidi, S. , Choi, M. , Wakimoto, H. , Ma, L. , Jiang, J. , Overton, J.D. et al. (2013) De novo mutations in histone‐modifying genes in congenital heart disease. Nature, 498, 220–223. 23665959 10.1038/nature12141 PMC3706629

