# Supplementary material for: Cyclin‐dependent kinase 13 is indispensable for normal mouse heart development
Source: J Anat. 2024 Nov 18;246(4):616–30. doi: 10.1111/joa.14175 (PMC11911135; doi:10.1111/joa.14175)
Supplement: Supplementary file 1 — Data S1: Supplementary Information. [file JOA-246-616-s001.pdf]

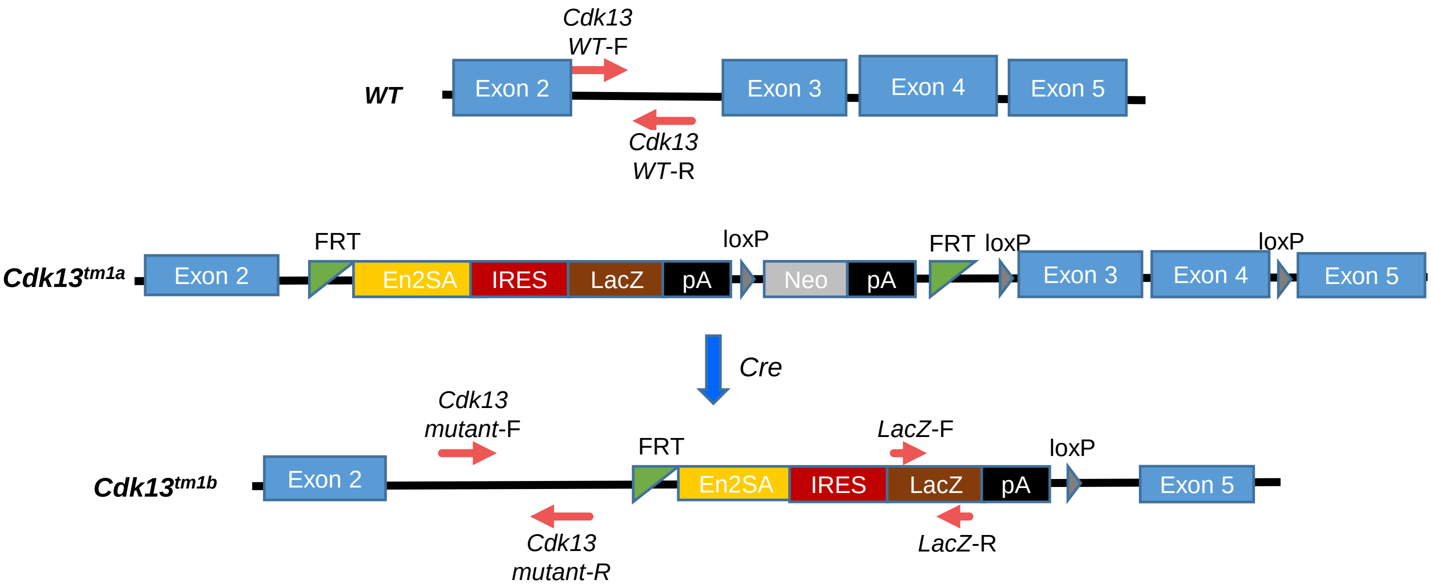

Supplementary Figure 1. Generation of tm1b allele from tm1a allele and *Cdk13*^{tm1b} mouse model. There is Cre-mediated replacement of critical exons with LacZ. The critical exons along with the two flanking loxP sites, FRT and neomycin cassette in tm1a allele are excised by Cre-recombinase to give rise to tm1b allele. In case of *Cdk13*^{tm1b}, critical exons excised are 3 and 4 and the allele contains LacZ cassette inserted into intron 2. WT denotes location of genotyping primers in intron 2 whereas *Cdk13*^{tm1b} indicates the location of primers used for detection of mutant allele located in intron 2, but the WT sequence finishes before the mutant reverse primer. Primers for detection of LacZ cassette in mutant allele are also shown. Primer sequences are given in Supplementary Table S1. En2SA, Engrailed 2 gene splice acceptor; FRT, flippase recognition target; Tm, target mutation; IRES, internal ribosome entry site; pA, polyA tail; WT, wild type.

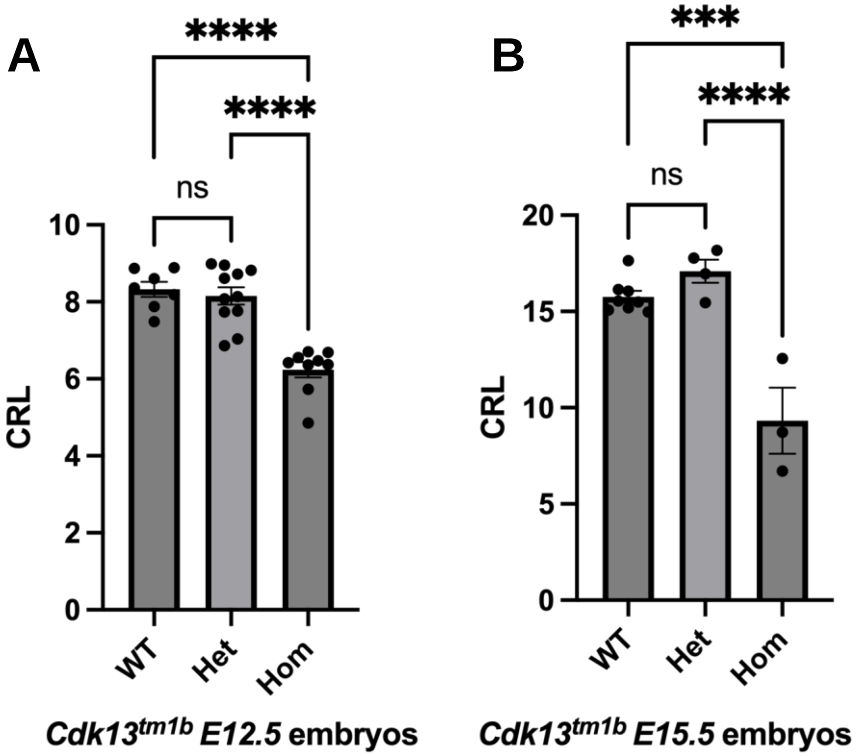

Supplementary Figure 2. Crown-rump length of *Cdk13^{tm1b}* mouse embryos . Crown-rump length (CRL) of the collected embryos was measured as the maximum distance between their cephalic and their caudal poles. Statistically significant reduction in CRL was noted in homozygous mouse embryos (n = 9 at E12.5 and 3 at E15.5) at both 12.5 (A) and E15.5 (B) compared to WT (n = 7 at E12.5 and 8 at E15.5) and heterozygotes (n = 11 at E12.5 and 4 at E15.5). Asterisk *** denotes $p < 0.005$ and **** denotes $P < 0.0005$; WT, wild type; Het, heterozygous; Hom, homozygous; ns, not significant.

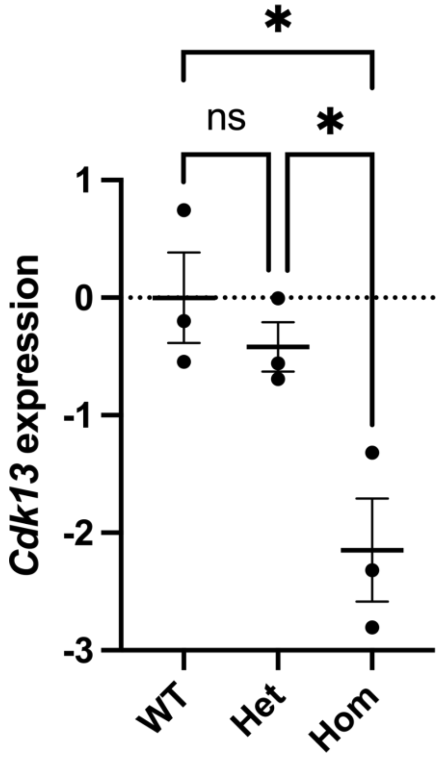

Supplementary Figure 3. Expression of *Cdk13 in* E10.5 *Cdk13^{tm1b}* mouse hearts by RT-qPCR. The $\log_2$FC of *Cdk13* (mean with SEM) in heterozygous (Het; *Cdk13$^{tm1b/+}$*) and homozygous (Hom; *Cdk13$^{tm1b/tm1b}$*) hearts collected at E10.5, compared to WT (*Cdk13$^{+/+}$*). Using one way-ANOVA and posthoc-Tukey test, no statistically significant difference is present in the level of *Cdk13* transcript in heterozygous compared to WT (P = 0.7023). In contrast the difference is significant in homozygous compared to WT (P = 0.0129) and heterozygous (P = 0.0330) hearts. n.s: not significant.

Table S1. Primers sequences used for genotyping and RT-qPCR assays.

Gene	Primer sequence (5' to 3')	Length (bp)	Efficiency (%) / r^2
**Primers for genotyping**			
***Cdk13* - Wild type**	Forward: GCTCTAAGGGCAACCTTGAA<br>Reverse: AGCTGGGAAGATGGTGTTGT	250	N/A
***Cdk13* - Mutant**	Forward: GCTCTAAGGGCAACCTTGAA<br>Reverse: TCGTGGTATCGTTATGCGCC	200	N/A
**LacZ**	Forward: GGTAAACTGGCTCGGATTAGGG<br>Reverse: TTGACTGTAGCGGCTGATGTTG	211	N/A
**Primers for RT-qPCR**			
Pgk1	Forward: GTCGTGATGAGGGTGGACTT<br>Reverse: AAGGACAACGGACTTGGCTC	126	108.9 / 0.99
Rpl4	Forward: GCCGCTGGTGGTTGAAGATAA<br>Reverse: CGTCGGTTTCTCATTTTGCCC	150	109 / 0.99
Cdk13	Forward: TGCCATGCTAGAGGAAAGGG<br>Reverse: CTGGTTCTGGTGTCAGCTCC	149	85.9 / 0.99
Sall4	Forward: ACCACGAAAGGCAACCTGAA<br>Reverse: TCCTTGGAAAACACCTCGGG	146	107.5 / 0.99
Vegfa	Forward: CCAAGATCCGCAGACGTGTA<br>Reverse: CTTTCCGGTGAGAGGTCTGG	172	103.5 / 0.99
Eln	Forward: ATTCCTGGGATTGGAGGCATTG<br>Reverse: ACTAAACCTCCAGCAGCTCCATA	104	93.32 / 0.99
Edn1	Forward: GGCCCAAAGTACCATGCAGA<br>Reverse: GATGGCCTCCAACCTTCGTA	127	105.8 / 0.969
Ednra	F: CACCTCAAACAGCGTCGAGA<br>R: TGCCAGGTTAATGCCGATGT	186	110 / 0.98

Bp, base pair; N/A, not applicable.

Cdk13$^{tm1b/tm1b}$

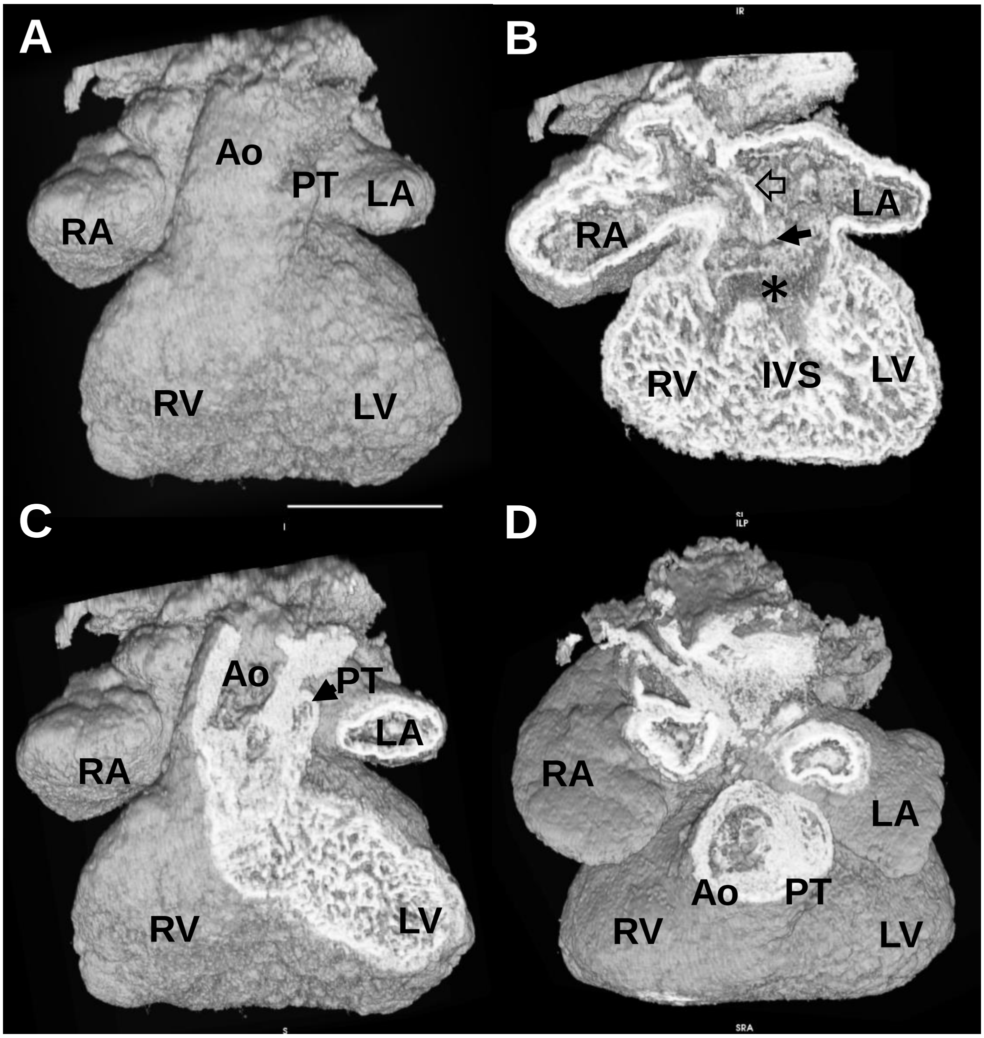

Supplementary Figure 4. Homozygous (*Cdk13*$^{tm1b/tm1b}$) E15.5 mouse heart with pulmonary stenosis, AVSD and DORV. Externally this homozygous heart is grossly abnormal with a cobblestone appearance (A). A four-chamber view shows that this *Cdk13*^{tm1b} homozygous heart has AVSD (B). The open arrow denotes the remaining atrial components, the black arrow the ostium primum defect and the back asterisk the VSD (B). Recesses can be seen in the interventricular septum (IVS), with the ventricular myocardium deeply trabeculated (B). A coronal view shows that the pulmonary trunk appears much smaller than the proximal aorta (C), which can also be seen on the external view (A), and on a superior view of the heart (D). This would result in pulmonary stenosis. However, both aortic and pulmonary valve cusps were discerned. This heart also had DORV with doubly committed subarterial single VSD with interconnected inlet and outlet components. Scale bar denotes 500 µm. Ao, aorta; AVSD, atrioventricular septal defect; DORV, double outlet right ventricle; LA:, left atrium; LV, left ventricle; PT, pulmonary trunk; RA, right atrium; RV, right ventricle; IVS, interventricular septum; VSD, ventricular septal defect.

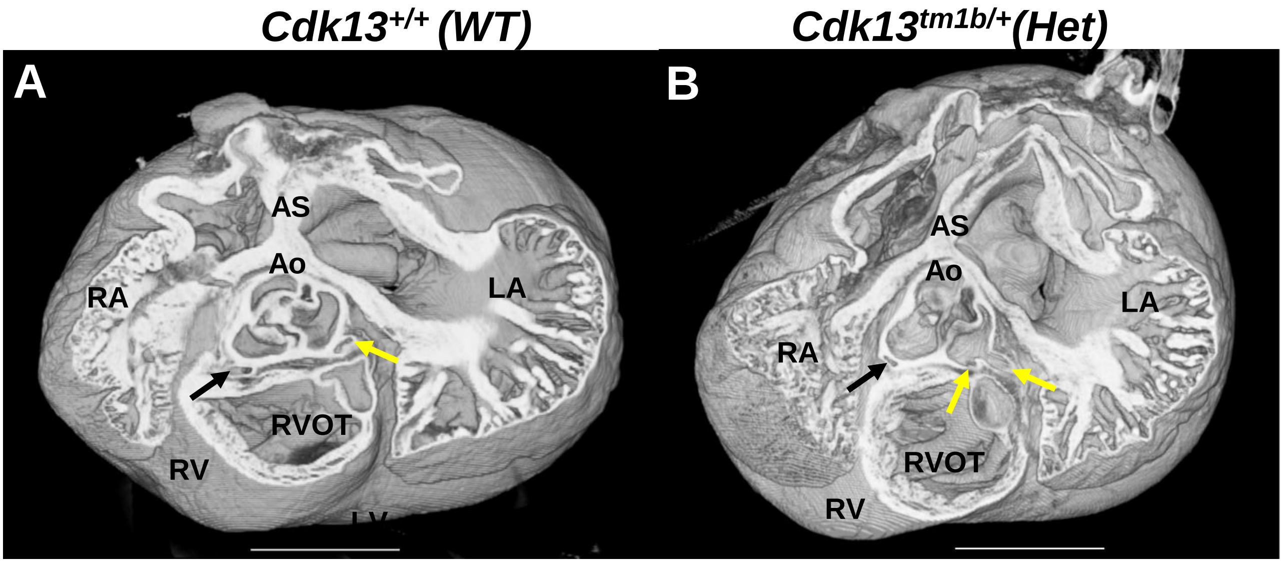

Supplementary Figure 5. Supernumerary coronary ostia in left aortic sinus in P6 heterozygous heart. Superior view axial sections of P6 neonatal hearts. Coronary ostia can be seen in a wild type control heart, with the two expected ostia (A) seen emerging from the aorta (from right coronary sinus denoted by a black arrow, and from left coronary sinus by a yellow arrow). In contrast, in a heterozygous heart two coronary ostia can be seen from the left aortic sinus (yellow arrows), instead of one, and a single ostium in right coronary sinus (black arrow). Scale bar denotes 1 mm. RA, right atrium; LA, left atrium; RV, right ventricle; AS, atrial septum; Ao, aorta; RVOT, right ventricular outflow tract.

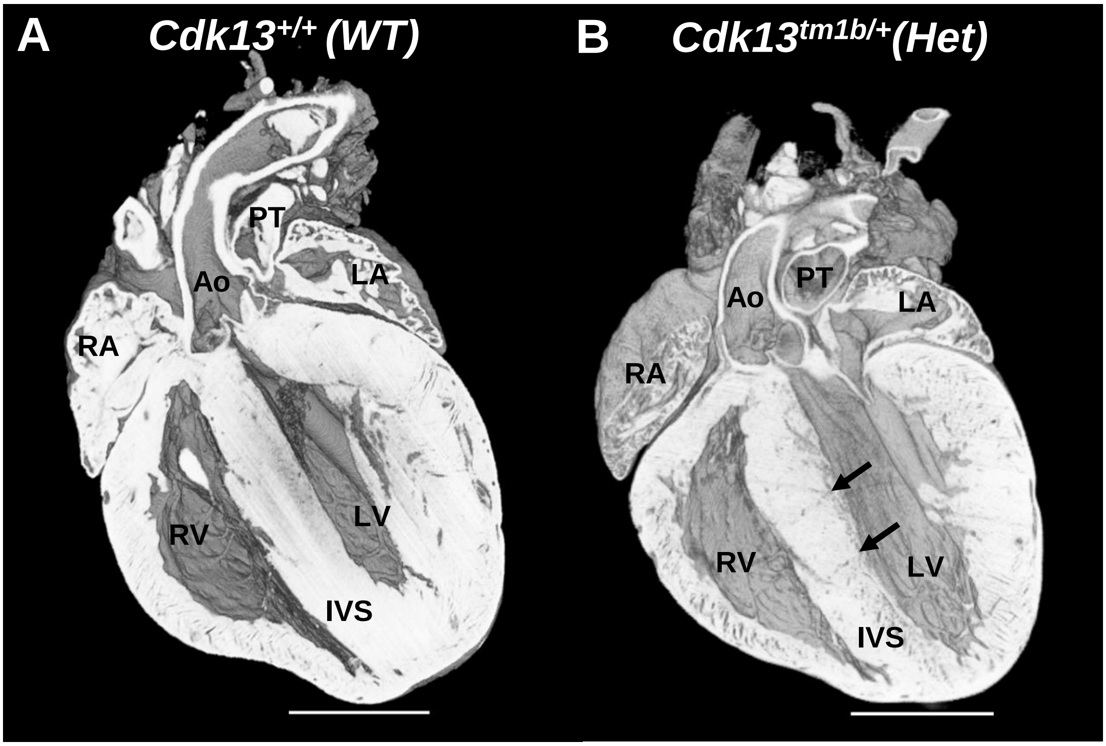

Supplementary Figure 6. Closing or closed muscular ventricular septal defects in P6 neonates. A *Cdk13*$^{tm1b/+}$ (Het) heart (B) having less dense areas in the muscular part of the ventricular septum (black arrows) compared to wild type (A). This is suggested to be closing/closed muscular VSDs. Scale bar denotes 1 mm. Ao, aorta; LA, left atrium; LV, left ventricle; PT, pulmonary trunk; RV, right ventricle; RA, right atrium; VS, ventricular septum; VSD ventricular septal defect; ET, wild type.
